# Evaluation of Economic and Health Outcomes Associated With Food Taxes and Subsidies: A Systematic Review and Meta-analysis

**DOI:** 10.1001/jamanetworkopen.2022.14371

**Published:** 2022-06-01

**Authors:** Tatiana Andreyeva, Keith Marple, Timothy E. Moore, Lisa M. Powell

**Affiliations:** 1 Department of Agricultural and Resource Economics, Rudd Center for Food Policy and Health, University of Connecticut, Hartford; 2 The Heller School for Social Policy and Management, Brandeis University, Waltham, Massachusetts; 3 Statistical Consulting Services, Center for Open Research Resources and Equipment, University of Connecticut, Storrs; 4 Health Policy and Administration, School of Public Health, University of Illinois Chicago, Chicago

## Abstract

**Importance:**

Fiscal policy is a promising approach to incentivizing better food choices and reducing the burden of chronic disease. To inform guidelines on using fiscal policies, including taxes and subsidies, to promote health, the World Health Organization commissioned a systematic review and meta-analysis of the worldwide literature on the outcomes of such policies for food products.

**Objective:**

To assess the outcomes of implemented food taxes and subsidies for prices, sales, consumption, and population-level diet and health.

**Data Sources:**

Eight bibliographic databases were searched for peer-reviewed literature and 14 data sources along with governmental websites were searched for grey literature that were published from database inception through June 1, 2020. There were no language and setting restrictions.

**Study Selection:**

Only primary studies of implemented food taxes and subsidies were considered for inclusion.

**Data Extraction and Synthesis:**

The Preferred Reporting Items for Systematic Reviews and Meta-analyses (PRISMA) reporting guideline was followed. A 3-level random-effects model was used to conduct a meta-analysis of sales and consumption outcomes of fruit and vegetable subsidies. Other outcomes were analyzed in a narrative synthesis.

**Main Outcomes and Measures:**

Study estimates in the meta-analysis were combined using a price elasticity measure for sales and consumption outcomes. Heterogeneity was assessed using the *I*^2^ statistic and τ^2^. Studies varied in how diet and health were measured.

**Results:**

A total of 54 articles were included in the systematic review, of which 15 studies were included in the meta-analysis. Most food subsidies targeted fruits and vegetables and populations with low income, whereas the evidence on food taxes was primarily from the nonessential energy-dense food tax in Mexico. Sales of subsidized fruits and vegetables increased significantly, with an estimated price elasticity of demand of −0.59 (95% CI, −1.04 to −0.13 [*P* = .02]; 95% prediction interval, −2.07 to 0.90; *I*^2^ = 92.4% [95% CI, 89.0%-94.8%; *P* < .001]), suggesting inelastic demand. There was no significant change in the consumption of subsidized fruits and vegetables, with an estimated price elasticity of demand of −0.17 (95% CI, −0.49 to 0.15 [*P* = .26]; 95% prediction interval, −1.01 to 0.67; *I*^2^ = 76.2% [95% CI, 54.3%-87.6%; *P* < .001]). Food excise taxes were associated with higher prices and reduced sales. Evidence was limited on the differential outcomes of food taxes and subsidies across subpopulations.

**Conclusions and Relevance:**

Results of this systematic review and meta-analysis indicated that fruit and vegetable subsidies were associated with a moderate increase in fruit and vegetable sales. Further research is warranted to understand the implications of food taxes and subsidies for population-level consumption, diet, and health outcomes.

## Key Points

**Question:**

What outcomes are associated with implemented food taxes and subsidies around the world?

**Findings:**

In this systematic review of 54 studies and meta-analysis of 15 studies, fruit and vegetable subsidies were associated with increased fruit and vegetable sales, with a price elasticity of −0.59, whereas changes in consumption were statistically insignificant. Food taxes were associated with higher prices and reduced sales of targeted products; evidence on other outcomes of food taxes and subsidies was limited.

**Meaning:**

Findings from this study suggest that fruit and vegetable subsidies to low-income populations are associated with increased sales of subsidized products, whereas food taxes may have the opposite results; additional research is needed to ascertain the implications of such policies for consumption, diet, and health outcomes.

## Teaser

This systematic review and meta-analysis evaluates the changes in food and beverage prices, sales, and consumption as well as in diets and health associated with food taxes and subsidies.

## Introduction

The prevalence of diet-related noncommunicable diseases (NCDs) has reached alarming proportions globally.^1^ Noncommunicable diseases account for more than 70% of deaths across the world, of which an estimated 40% can be attributed to dietary factors.^2^ Premature deaths and high health care costs could be avoided by addressing major dietary deficiencies, including low intake of fruits, vegetables, and whole grains as well as excessive consumption of added sugars, saturated fats, and sodium. In response to unfavorable dietary and health patterns, many countries have been considering the adoption of fiscal policies (taxes and subsidies) to incentivize better food choices.^3,4^ The World Health Organization (WHO) supports the “consideration of economic tools, where justified by evidence, which may include taxes and subsidies, that create incentives for behaviors associated with improved health outcomes, as appropriate within the national context.”^4^ It is critical to assess the evidence on the extent to which price interventions have implications for food consumption, diet quality, and health outcomes.

Economic theory suggests that consumer demand responds inversely to price changes; thus, fiscal policy can encourage consumer behavior changes by shifting relative prices up (through taxation) or down (through subsidies). Subsidies can be used to increase the demand for healthful products that are underconsumed. Imposing excise taxes on products with known public health consequences, such as tobacco and alcohol, has long been a strategy to discourage smoking and excessive drinking; more recently, taxes have been imposed on sugary beverages and selected foods and nutrients associated with diet-related health risks.^5,6^

There are new data available on the outcomes of fiscal policies (taxes and subsidies) for foods and beverages. Previous systematic reviews^7,8,9,10,11^ have suggested that price interventions targeting healthy and unhealthy foods could alter consumer choices in favor of better nutrition. This literature included estimates from modeling studies and demand analyses.^7,8,9,10,11^

The present systematic review and meta-analysis of the literature on real-world food taxes and subsidies aimed to assess the outcomes associated with implemented food taxes and subsidies for prices, sales, consumption, and population-level diet and health. This study was part of a broader systematic review of the effectiveness of fiscal and pricing policies for foods and nonalcoholic beverages that was commissioned by the WHO to inform guidelines for its member states on the development of such policies to promote healthy diets.

## Methods

### Search Strategy

In this systematic review and meta-analysis (PROSPERO, registration CRD42019139426), we included peer-reviewed and grey literature from all countries and published in all languages from database inception through June 1, 2020. We adhered to the Preferred Reporting Items for Systematic Reviews and Meta-analyses (PRISMA) reporting guideline.^12^

The search was guided by the Population, Intervention, Comparison, and Outcome framework, defined by the WHO Nutrition Guidance Expert Advisory Group Subgroup on Policy Actions, and included outcomes that were deemed as critical: price changes, direct and substitution-associated sales (including both store sales and household purchases) of taxed or subsidized products and their substitutes, consumption (direct and substitution changes), and dietary intake (eg, energy, total food and/or nutrient intake, and nutritional quality). Outcomes that were deemed as important by the Nutrition Guidance Expert Advisory Group subgroup were product changes (eg, portion size and food reformulation), unintended consequences (eg, jobs and cross-border shopping), body weight status or body mass index (BMI), diet-related NCDs, undernutrition, and pregnancy outcomes.

For peer-reviewed literature, we searched 8 bibliographic electronic databases, including Business Source Complete, Cochrane Central Register of Controlled Trials, Cochrane Database of Systematic Reviews, Cumulative Index to Nursing and Allied Health Literature Plus With Full Text, EconLit, PsycInfo, PubMed, and Scopus. For grey literature, we searched these 14 sources: Directory of Open Access Journals, EconPapers, Database of Promoting Health Effectiveness Reviews, Trials Register of Promoting Health Interventions, Google Scholar, Healthevidence.org, Health Services Research Projects in Progress, National Bureau of Economic Research, PDQ-Evidence for Informed Health Policymaking, ProQuest Dissertations and Theses Database, Social Science Research Network eLibrary, WHO Global Index Medicus, WHO International Clinical Trials Registry Platform, and WorldWideScience.org. Websites of relevant organizations and government agencies were also searched. References in relevant systematic reviews and papers that were selected for data extraction were scanned for additional studies.

The search strategy and results are detailed in eAppendix 1 in the Supplement. A librarian at the University of Connecticut assisted in developing the search strategy.

### Eligibility Criteria

We assessed current or past fiscal and pricing policies for food products, including subsidies of any type (eg, discounts, vouchers, and coupons), taxes of any nature (eg, excise and sales), and fiscal or pricing interventions targeting any food products or nutrients. Beverages were assessed separately and were not included in this study. We did not assess policies that could affect consumer prices but were not direct fiscal or pricing policies, such as import tariffs, agricultural subsidies, and cash transfer and in-kind transfer programs. Policy implementation was compared with nonimplementation of a tax or a subsidy. We hypothesized that lower prices of subsidized healthier foods would encourage their sales and consumption and that taxes would lead to higher prices, lower sales, and lower consumption of taxed foods.

The study populations included children (aged <18 years) and adults (aged ≥18 years) from any country and setting. Only primary research or reports were considered for inclusion; editorials, commentaries and reviews, modeling or simulation studies, and cost-effectiveness articles were excluded. Experimental studies were included if they assessed real-world fiscal policies. Studies with the following designs were eligible: randomized trial, interrupted time series, controlled and uncontrolled before-and-after study, quasi-experimental study, cross-sectional analysis using propensity score matching, difference-in-differences method or fixed-effect analysis, longitudinal analysis using fixed effects, or ecological analysis.

### Data Collection and Extraction

Two of us (T.A. and K.M.) independently screened titles and abstracts, assessed full-text articles, completed data extraction, and assessed study quality. Any disagreement was resolved through consensus and discussion with another author (L.M.P.).

Given that all tax and most subsidy studies were nonexperimental, we evaluated study quality using a new tool that was adapted from a systematic review of beverage taxes^13^ and informed by a risk-of-bias tool for nonrandomized studies of interventions (ROBINS-I tool; Cochrane).^14^ This new quality-of-study tool (eTable 1 in the Supplement) was developed to capture multiple components of policy evaluations, including the study design, validity of measures, sample selection, and adequate control for confounders. Assessment was done at the outcome level rather than study level because study designs could vary across the outcomes within 1 article. Using 7 questions to evaluate the methodological rigor and data limitations, we assigned a score of low, medium, or high quality to each outcome in every reviewed paper.

We selected 1 estimate per outcome except when a study examined more than 1 policy or used multiple data sets per outcome. Estimated changes across the postintervention period were selected; alternatively, the latest changes reported after the intervention were used. Whenever possible, estimated relative changes were extracted; when only absolute changes were reported, they were converted into relative changes by dividing both the estimated changes and CIs by the baseline estimates. Volumetric measures were selected over measures of frequency or expenditure. In studies that presented results of more than 1 model specification, we used results from the investigators’ preferred model; otherwise, the most fully controlled model was chosen.

Investigators were contacted by email if we needed to request missing data. They were not contacted for studies that did not provide statistical testing of estimates.

### Statistical Analysis

The synthesis of results proceeded in 2 stages. When a meta-analysis was feasible, we used it on results of studies with complete data. Studies with missing data, that lacked statistical testing, or with duplicate results (ie, multiple publications of the same studies) were excluded from the meta-analysis and included in the narrative analysis. For outcomes with few available studies (<7) or with high heterogeneity across types of policies and measures, we analyzed the studies in a narrative synthesis only.

Study estimates in the pooled meta-analysis were combined using a price elasticity measure for sales and consumption and calculated as a percentage change in demand over a percentage change in price. Postintervention percentage change in price was calculated as a percentage discount provided by the subsidy. Elasticities and their CIs were computed from relative changes in demand by dividing the estimated percentage changes in demand and corresponding CIs by the percentage changes in price. eAppendix 2 in the Supplement provides details on the computation of the price elasticity measures.

The meta-analysis was conducted to generate pooled effect size estimates using restricted maximum likelihood for estimating τ^2^. We used Hartung-Knapp–adjusted, 3-level random-effects models to account for the expected high between-study heterogeneity and for some studies with more than 1 effect size (ie, effect sizes were clustered within studies).^15^ The proportion of the variance explained was assessed using the *I*^2^ statistic.^16^ In addition, 95% prediction intervals were estimated to identify the expected range of true effect sizes in similar studies, providing a measure that accounts for both the variance in the estimated outcome and the between-study heterogeneity (τ^2^).^17,18^

For the outcomes in the meta-analysis, publication bias was assessed using the Egger test.^19^ The meta-analysis was conducted in R, version 4.1.0^20^ using the meta package version 4.19,^21^ with the 95% prediction intervals calculated using the metafor package^22^ and auxiliary functions from the dmetar package, version 0.09.000^23^ (R Foundation for Statistical Computing). In the narrative synthesis, the results were aggregated by the direction of estimated results (increase or decrease) and statistical significance of the estimates.

## Results

**Figure 1.**
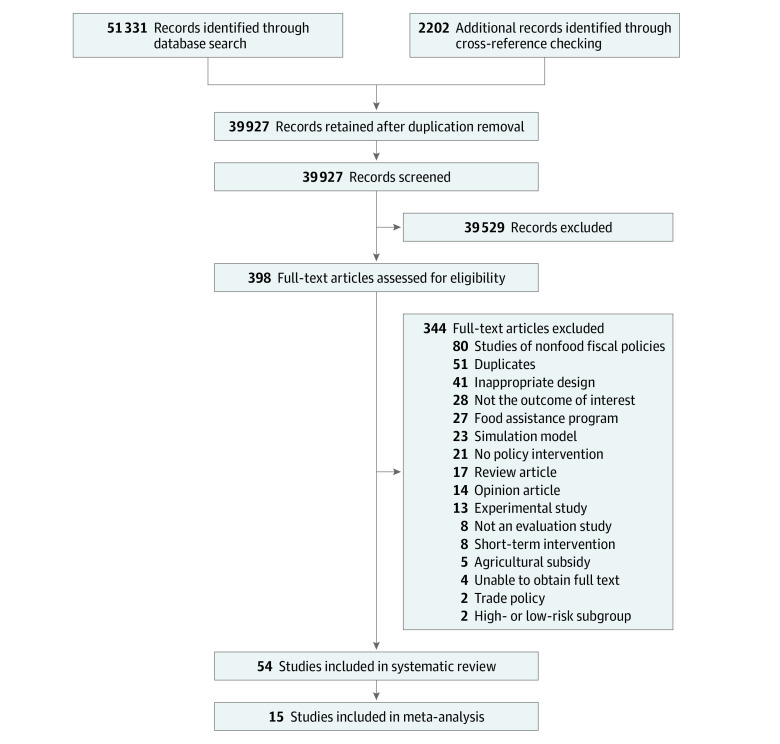
PRISMA Diagram of Study Selection

A total of 39 927 unique titles were retrieved for abstract and title screening, from which 398 titles were selected for full-text screening (Figure 1). We identified 54 articles^24,25,26,27,28,29,30,31,32,33,34,35,36,37,38,39,40,41,42,43,44,45,46,47,48,49,50,51,52,53,54,55,56,57,58,59,60,61,62,63,64,65,66,67,68,69,70,71,72,73,74,75,76,77^ that met the inclusion criteria; 43 of which were peer-reviewed studies, and 11 were reports, dissertations, or working papers. No studies were found on pricing policies (eg, minimum pricing floor) for food products.

Most studies (n = 35 of 54 [64.8%]) evaluated the outcomes of food subsidies, including 30 articles focusing on fruit and vegetable subsidies,^28,29,30,31,34,35,36,39,43,45,47,48,50,51,52,56,57,58,60,62,63,65,66,67,69,71,72,75,76,77^ 3 on healthy food subsidies,^26,27,73^ and 2 on staple food (such as pulses and grains) subsidies.^40,41^ Most studies on fruit and vegetable subsidies were conducted in the US.^28,29,30,31,39,43,45,48,50,51,52,56,57,58,62,63,65,66,67,69,71,72,75,76,77^ With 4 exceptions,^26,27,60,73^ all subsidy interventions targeted populations with low income, with US-based studies often focusing on participants in federal food assistance programs or people whose income made them eligible to receive assistance.

There were 19 studies (35.2%) on food taxes, including 10 on the nonessential energy-dense food tax in Mexico,^24,25,32,38,42,49,59,64,68,74^ 5 on the now-repealed saturated fat tax in Denmark,^37,44,54,55,70^ 3 on state sales taxes in the US,^46,53,61^ and 2 on the public health product tax in Hungary.^33,44^ All studies on food taxes used a nonexperimental design (n = 19 of 54 [35.2%]), whereas a third of fruit and vegetable subsidy articles were randomized trials (n = 10 of 30 [33.3%]).^31,45,48,50,56,58,63,65,66,76^

The most common outcome of the reviewed studies was sales (n = 28 [51.9%]),^24,28,31,32,33,37,38,40,44,45,47,48,51,53,54,55,58,59,64,65,66,67,70,72,73,74,75,76^ which was followed by consumption (n = 19),^26,27,29,30,31,34,39,40,43,50,52,56,57,58,62,63,69,71,75^ prices (n = 11),^24,25,38,42,44,51,54,55,60,68,77^ sales of substitute products (n = 12),^24,32,33,38,45,53,58,64,67,70,73,74^ consumption of substitute products (n = 6),^26,27,31,39,50,63^ dietary intake (n = 5),^31,34,40,50,63^ BMI (n = 5),^27,35,46,50,61^ diet-related NCDs (n = 2),^34,36^ undernutrition (n = 2),^35,41^ and unintended consequences (n = 2).^49,59^ No studies assessed product changes or pregnancy outcomes.

The study quality was highly variable (eTable 2 in the Supplement). Most studies of subsidized food consumption (n = 13 of 19 [68.4%]) were rated as low quality, reflecting their use of small convenience samples and limited subjective measures. Only 4 studies on consumption, all of which were randomized trials,^31,50,56,63^ were ranked as high quality. In contrast, studies of food sales were usually high quality (n = 19 of 28 [67.9%]), with only 3 studies^38,44,59^ ranked as low quality. Studies of other outcomes were of variable quality.

Results from all studies on food taxes^24,25,32,33,37,38,42,44,46,49,53,54,55,59,61,64,68,70,74^ and subsidies other than for fruits and vegetables^26,27,40,41,73^ were included in the narrative synthesis. A meta-analysis was not conducted for these results because of the low number of studies and/or the large heterogeneity across policies and measures. Of the studies on fruit and vegetable subsidies, only 2 outcomes (sales and consumption of subsidized products) had sufficient data for a meta-analysis; other outcomes were analyzed in the narrative synthesis. Fifteen of 54 studies (27.8%), which consisted of randomized trials^31,45,50,58,63,65,66,76^ and non-randomized trials,^28,34,39,62,67,72,75^ were included in the meta-analysis. For the 2 outcomes of fruit and vegetable subsidies in the meta-analysis, 11 articles^29,30,43,47,48,51,52,56,57,69,71^ were included in the narrative synthesis only because they did not have sufficient data for inclusion in the meta-analysis or they were results of the already included studies.

### Meta-analysis

**Figure 2.**
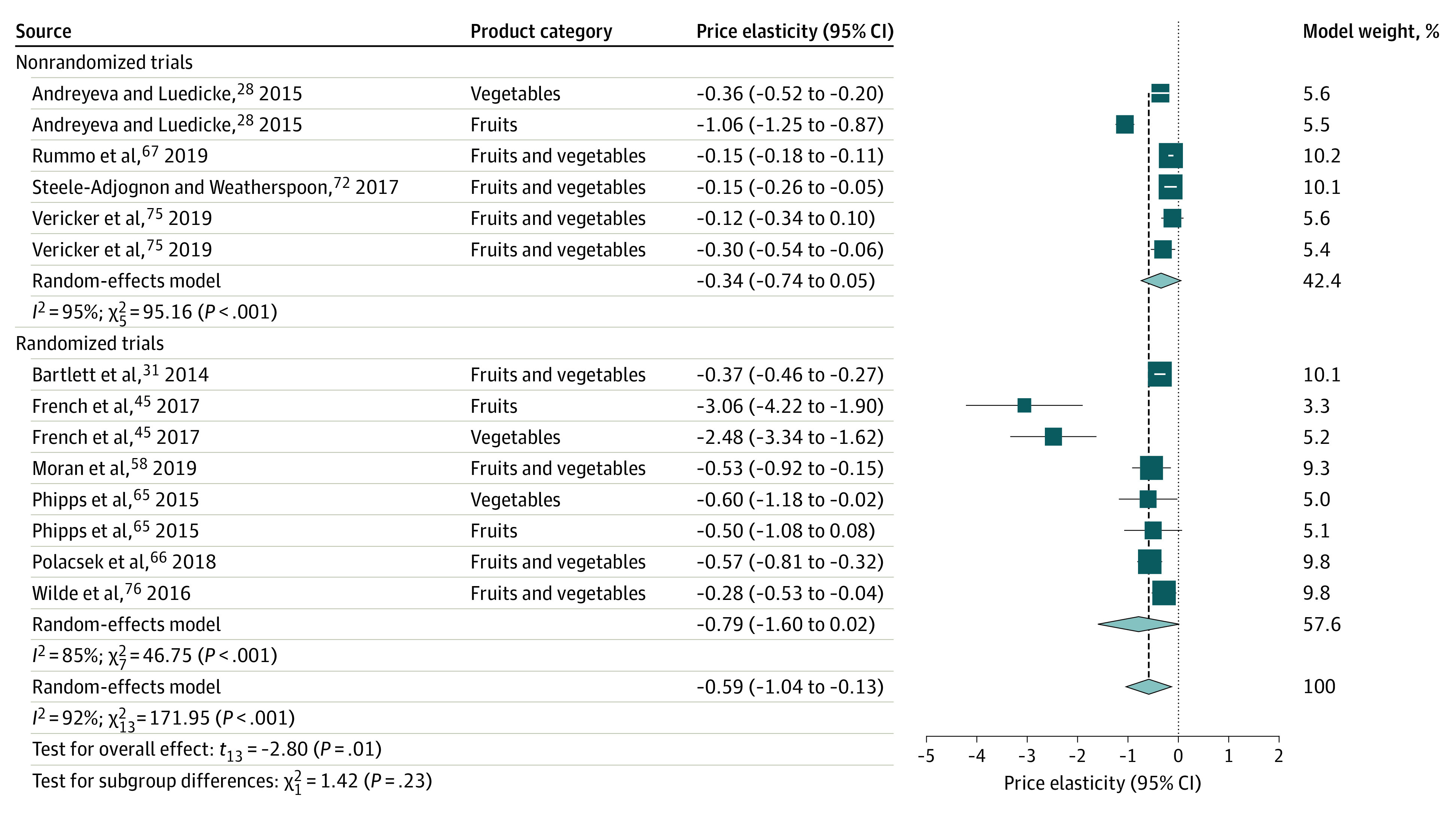
Forest Plot of Price Elasticity of Demand: Fruit and Vegetable Sales After Subsidies The elasticity measures the percentage change in sales from a 1% change in price. A negative value represents an inverse association between price and sales. A subsidy is a decrease in price, so a subsidy (lower price) is expected to be associated with an increase in sales. Proportionally sized squares represent the weight of each study and diamonds show the overall effect for randomized vs nonrandomized trials separately and combined. Horizontal lines represent 95% CIs. Vertical dashed line indicates point estimate of overall pooled effect.

Summary results from the meta-analysis are presented in Table 1. A total of 15 studies were included in the meta-analysis. Fruit and vegetable subsidies were associated with a significant increase in fruit and vegetable sales, with a price elasticity of demand of −0.59 (95% CI, −1.04 to −0.13 [*P* = .02]; 95% prediction interval, −2.07 to 0.90; *I*^2^ = 92.4% [95% CI, 89.0%-94.8%; *P* < .001]) (Table 1; Figure 2). Price elasticity of −0.59 suggested that a subsidy that lowered the price of fruits and vegetables by 10% was associated with an increase in mean sales by 5.9%, indicating inelastic demand for fruits and vegetables among populations with low income. Subgroup analyses of randomized and nonrandomized trials found no significant between-group difference (*Q* statistic = 1.42; *P* = .23), with a pooled estimate of price elasticity of −0.79 (95% CI, −1.60 to 0.02 [*P* = .054]; *I*^2^ = 85.0%) for randomized trials and −0.34 (95% CI, −0.74 to 0.05 [*P* = .08]; *I*^2^ = 94.7%) for nonrandomized trials (Table 1; Figure 2).

**Table 1.** Meta-analysis of Sales and Consumption Outcomes After Fruit and Vegetable Subsidies

Outcome	No. of estimates	No. of articles	Pooled estimate (95% CI)	*P* value	95% Prediction interval	Heterogeneity *Q*	*P* value	Heterogeneity *I*^2^, % (95% CI)	Publication bias
Sales: price elasticity	14	10	−0.59 (−1.04 to −0.13)	.02	−2.07 to 0.90	172	<.001	92.4 (89.0 to 94.8)	Yes
Randomized trial	8	6	−0.79 (−1.60 to 0.02)	.05		47		85.0 (72.3 to 91.9)	
Nonrandomized trial	6	4	−0.34 (−0.74 to 0.05)	.08		95		94.7 (91.0 to 96.9)	
Consumption: price elasticity	9	7	−0.17 (−0.49 to 0.15)	.26	−1.01 to 0.67	34	<.001	76.2 (54.3 to 87.6)	None
Randomized trial	4	3	−0.45 (−1.50 to 0.59)	.26		22		86.3 (66.8 to 94.4)	
Nonrandomized trial	5	4	−0.02 (−0.20 to 0.15)	.72		9		56.9 (0 to 84.0)	

**Figure 3.**
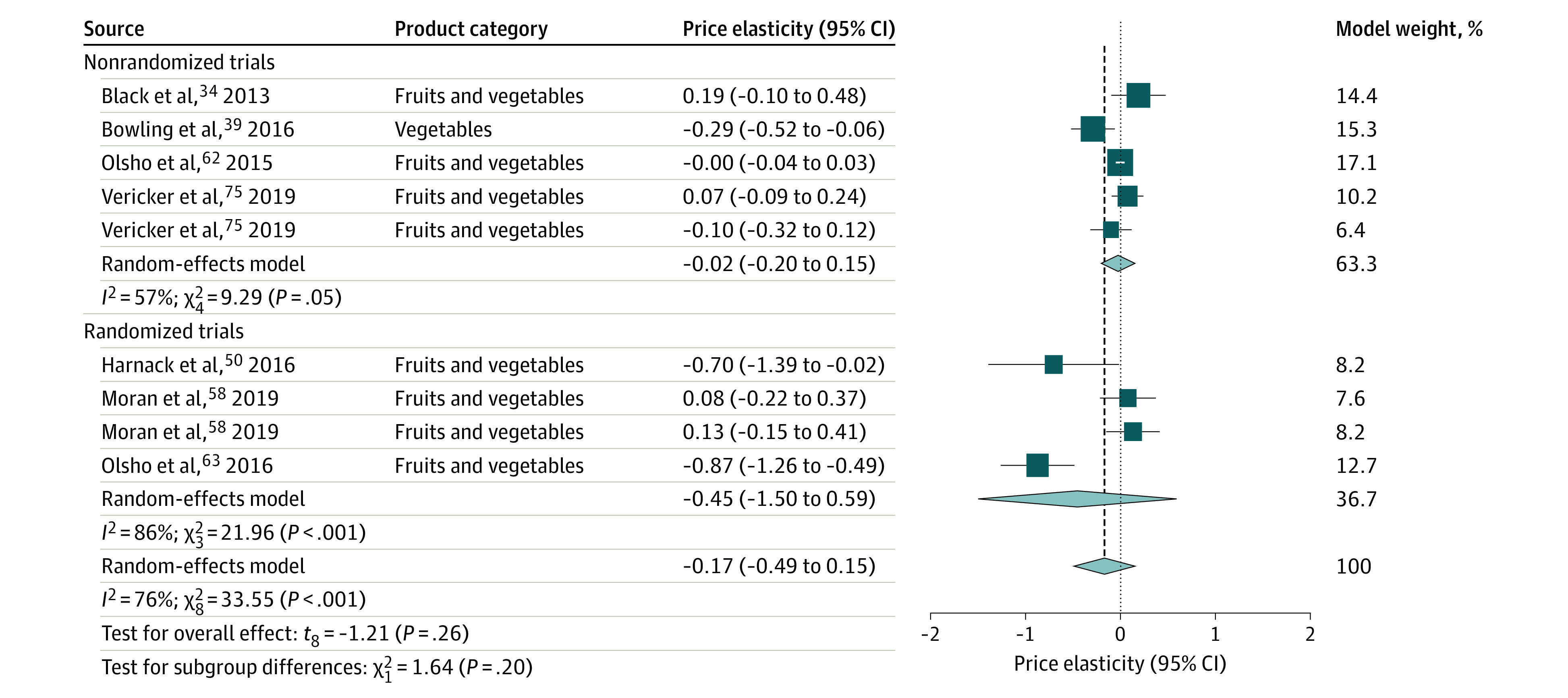
Forest Plot of Price Elasticity of Demand: Fruit and Vegetable Consumption After Subsidies The elasticity measures the percentage change in sales from a 1% change in price. A negative value represents an inverse association between price and sales. A subsidy is a decrease in price, so a subsidy (lower price) is expected to be associated with an increase in sales. Proportionally sized squares represent the weight of each study and diamonds show the overall effect for randomized vs nonrandomized trials separately and combined. Horizontal lines represent 95% CIs. Vertical dashed line indicates point estimate of overall pooled effect.

Consumption of subsidized fruits and vegetables did not change significantly, with a pooled estimate of price elasticity of demand of −0.17 (95% CI, −0.49 to 0.15 [*P* = .26]; 95% prediction interval, −1.01 to 0.67; *I*^2^ = 76.2% [95% CI, 54.3%-87.6%; *P* < .001]) (Table 1; Figure 3). Subgroup analyses of randomized and nonrandomized trials on consumption showed no between-group difference, and the pooled estimates from the meta-analysis of randomized and nonrandomized trials were both not statistically significant. The number of studies within each subgroup was small.

### Sensitivity Analysis in Meta-analysis

No outlier studies were identified in the meta-analysis of consumption outcomes of fruit and vegetable subsidies. One study^45^ with 2 estimates was an outlier in the sales meta-analysis; its removal did not substantially change heterogeneity (*I*^2 ^>75%) but was associated with a reduced pooled estimate of price elasticity (−0.39; 95% CI, −0.58 to −0.21; *P* < .01).

### Publication Bias

Egger test results suggested no evidence of publication bias in studies on consumption (β_0_ = −0.98; 95% CI, −2.71 to 0.76; *P* = .31). In contrast, we found a significant negative intercept for studies on fruit and vegetable sales (β_0_ = −3.28; 95% CI, −5.17 to −1.38; *P* < .01), which was suggestive of publication bias. These results were affected by studies with large effect sizes and/or high precision, which in turn had a substantial role in the estimation of the intercept given the small overall sample size (eFigure in the Supplement).

### Narrative Synthesis

Studies on fruit and vegetable subsidies that could not be included in the meta-analysis had results similar to the 2 outcomes included in the meta-analysis. Subsidies were associated with significantly higher sales of fruits and vegetables,^47,48,51^ and mixed results were found for consumption (ie, no change in some studies^29,30,69,71^ and increases in others^31,43,52,56,57^) (Table 2).

**Table 2.** Summary of Results From Narrative Synthesis

Fiscal policy	Location	Outcomes	No. of studies	Direction and statistical significance of estimated outcomes
Meta-analysis of outcomes in narrative synthesis				
Fruit and vegetable subsidy	UK; US	Sales (direct)	3	Significant increase: Griffith et al,^47^ 2018; Grindal et al,^48^ 2016 Marginally statistically significant increase at the 10% level: Henderson,^51^ 2020
	US	Consumption (direct)	9	Significant increase: Bartlett et al,^31^ 2014; Durward et al,^43^ 2019; Herman et al,^52^ 2008; Klerman et al,^56^ 2014; Lindsay et al,^57^ 2013 No significant change: Anliker et al,^29^ 1992; Atoloye,^30^ 2019; Savoie-Roskos et al,^69^ 2016; Smith,^71^ 2017
Analysis of policies in narrative synthesis only				
Nonessential energy-dense food excise tax	Mexico	Price changes	5	Significant increase: Aguilar Esteva et al,^24^ 2019; Aguilera Aburto et al,^25^ 2017; Colchero et al,^42^ 2017; Salgado and Ng,^68^ 2019 Increase, no statistical testing: Bonilla-Chacin et al,^38^ 2016
		Sales (direct)	6	Significant decrease: Aguilar Esteva et al,^24^ 2019; Batis et al,^32^ 2016; Pedraza et al,^64^ 2018; Taillie et al,^74^ 2017 Mixed results: de Jesús Moreno Neri et al,^59^ 2016 No significant change: Bonilla-Chacin et al,^38^ 2016
		Sales (substitution)	5	Significant increase: Aguilar Esteva et al,^24^ 2019 Mixed results: Bonilla-Chacin et al,^38^ 2016; Pedraza et al,^64^ 2018 No significant change: Batis et al,^32^ 2016; Taillie et al,^74^ 2017
		Unintended consequence: unemployment	2	Significant decrease: Guerrero-López et al,^49^ 2017 Increase, no statistical testing: de Jesús Moreno Neri et al,^59^ 2016
Candy and snacks state sales tax	US	BMI	2	No significant change: Gordes,^46^ 2016; Oaks,^61^ 2005
		Sales (direct)	1	No significant change: Hoy,^53^ 2017
		Sales (substitution)	1	Significant increase: Hoy,^53^ 2017
Saturated fat excise tax	Denmark	Price changes	3	Significant increase: Jensen and Smed,^54^ 2013; Jensen et al,^55^ 2016 Increase, no statistical testing: ECSIPC,^44^ 2014
		Sales (direct)	5	Significant decrease: Jensen and Smed,^54^ 2013; Jensen et al,^55^ 2016 Decrease, no statistical testing: Bødker et al,^37^ 2015; ECSIPC,^44^ 2014; Smed et al,^70^ 2016
		Sales (substitution)	1	Mixed results, no statistical testing: Smed et al,^70^ 2016
Snacks and confectionary excise tax	Denmark; Finland	Price changes	1	Increase, no statistical testing: ECSIPC,^44^ 2014
		Sales (direct)	1	Decrease, no statistical testing: ECSIPC,^44^ 2014
Public health product tax	Hungary	Price changes	1	Increase, no statistical testing: ECSIPC,^44^ 2014
		Sales (direct)	2	Significant decrease: Bíró,^33^ 2015 Decrease, no statistical testing: ECSIPC,^44^ 2014
		Sales (substitution)	1	No significant change: Bíró,^33^ 2015
Healthy foods subsidy	South Africa	Consumption (direct)	2	Significant increase: An and Sturm,^26^ 2017; An et al,^27^ 2013
		Consumption (substitution)	2	Significant decrease: An and Sturm,^26^ 2017; An et al,^27^ 2013
		Sales (direct)	1	Significant increase: Sturm et al,^73^ 2013
		Sales (substitution)	1	Significant decrease: Sturm et al,^73^ 2013
		BMI	1	No significant change: An et al,^27^ 2013
Staple foods subsidy	India	Consumption (direct)	1	Significant increase: Chakrabarti et al,^40^ 2018
		Sales (direct)	1	Significant increase: Chakrabarti et al,^40^ 2018
		Dietary intake	1	Significant increase: Chakrabarti et al,^40^ 2018
		Undernutrition	1	No significant change: Chakrabarti et al,^41^ 2019
Fruit and vegetable subsidy	US	Consumption (substitution)	4	Mixed results: Bartlett et al,^31^ 2014; Harnack et al,^50^ 2016; Olsho et al,^63^ 2016 Significant decrease: Bowling et al,^39^ 2016
	US; Australia	Dietary intake	4	No significant change: (Black et al,^34^ 2013; Harnack et al,^50^ 2016) Mixed results: Bartlett et al,^31^ 2014 Significant increase: Olsho et al,^63^ 2016
	US; Latvia	Price changes	3	Significant decrease: Nipers et al,^60^ 2019 Mixed results: Henderson,^51^ 2020; Zenk et al,^77^ 2014
	US	Sales (substitution)	3	No significant change: Moran et al,^58^ 2019; Rummo et al,^67^ 2019 Mixed results: French et al,^45^ 2017
	Australia	Diet-related NCDs	2	Significant increase: Black et al,^36^ 2014 Mixed results: Black et al,^34^ 2013
	US; Australia	BMI	2	No significant change: Black et al,^35^ 2013; Harnack et al,^50^ 2016
	Australia	Undernutrition	1	Mixed results: Black et al,^34^ 2013

Abbreviations: BMI, body mass index; ECSIPC, European Competitiveness and Sustainable Industrial Policy Consortium; NCD, noncommunicable disease.

The evidence on the nonessential energy-dense food tax in Mexico had consistent results of increased prices^24,25,42,68^ and reduced sales of taxed products.^24,32,64,74^ There were mixed findings on how purchases of substitutes or untaxed products changed. Some studies reported no significant changes in untaxed food sales,^32,74^ and results in the other studies varied across measures.^38,64^ For the unintended consequences outcome, 2 studies examined unemployment: 1 reported a decreasing pattern of national unemployment,^49^ and 1 small bakery study^59^ reported lower employment but did not provide statistical testing. No studies were available to allow an assessment of the association of the nonessential energy-dense food tax with consumption of taxed products, dietary intake, BMI, and NCDs.

Similar findings for sales and prices were seen in the association with other food excise taxes, including a saturated fat tax in Denmark (which was repealed), a public health product tax in Hungary, and confectionary and snack taxes in several countries (Table 2). A major limitation in several studies was the lack of statistical testing.^37,44,70^ Besides sales and prices, no other outcomes were evaluated for their association with excise taxes on food or saturated fat. Two studies^46,61^ of the US-based sales taxes reported no significant changes in BMI associated with these small taxes.

In contrast to the studies on taxes, there were only a few studies on price changes for subsidies,^51,77^ and their results were mixed or were not significant, with 1 study showing lower market prices.^60^ Dietary quality changes were assessed in 4 studies on fruit and vegetable subsidies, including 1 randomized trial that found a statistically significant increase in dietary quality but no change in energy intake^31,63^ as well as 1 randomized trial^50^ and a nonrandomized trial^34^ that found no detectable changes in diet. A UK-based study^47^ reported an association of fruit and vegetable subsidies with increased sales of fruits and vegetables and improvements in the nutritional composition of the household shopping baskets. The study also found increases in the proportion of households meeting their recommended dietary reference intakes and higher consumption of nutrients that are important for child development (fiber; beta carotene; vitamins C, D, and E; potassium; iron; and zinc).^47^ There were no changes in BMI^35,50^ and limited data on NCD measures in studies on fruit and vegetable subsidies, including evidence of a substantial increase in the mean red blood cell folate *z* score for children^36^ and elevated levels in 3 of 9 examined biomarkers at the 12-month follow-up (such as beta cryptoxanthin, vitamin C, and lutein and zeaxanthin).^34^

In studies on subsidies for products other than fruits and vegetables, there was evidence of increased sales and/or consumption of subsidized foods.^26,27,40,73^ The association between the discount and the relative change in sales or consumption of subsidized products did not appear to be linear in 2 studies.^26,27^ For example, a 10% discount on healthy food purchases was associated with higher consumption of fruits and vegetables by 0.382 daily servings, whereas a 25% subsidy was associated with a 0.636 serving increase.^27^ Purchases of less healthy foods decreased after the healthy food discount, but the reduction was small.^73^ Dietary intake was assessed in 1 study of subsidized pulse purchases and found a small but significant increase in protein intake from pulses (1.38 g/d/household).^40^ There were no significant changes in BMI^27^ or undernutrition.^41^

### Subgroup Analyses

Only a fraction of studies on food taxes included any subgroup comparisons.^32,38,46,64,74^ For the nonessential energy-dense food tax in Mexico, the evidence suggested that sales of taxed foods declined more for households with low socioeconomic status.^32,64^ No studies evaluated the outcomes of the Danish saturated fat tax across subpopulations, and no data were reported on the heterogeneity of consumer responses to excise taxes on various nonessential foods (eg, snacks and confectionary). Just 1 study of state sales snack taxes considered subgroup analyses and found an inverse association between snack taxes and BMI for high-school graduates only.^46^

With 4 exceptions, studies on subsidies targeted families with low income.^26,27,60,73^ Differential changes across other sociodemographic characteristics were typically not provided, with rare exceptions. The Healthy Incentives Pilot in the US examined the outcomes of fruit and vegetable subsidies by sex and work status but found no significant differences among these groups.^31,56^ A study on fruit and vegetable subsidies in Australia^36^ reported regional differences. Few studies, of which none were in the US, focused specifically on children.^34,35,36^

## Discussion

In this systematic review and meta-analysis of worldwide literature on the outcomes of implemented food subsidies and taxes, we found that most studies on fiscal policies for foods focused on changes in sales, with evidence of changes in food consumption associated with food taxes completely lacking. Almost all implemented food subsidies targeted populations with low income, and most of these programs promoted fruits and vegetables. Some evidence regarding fruit and vegetable subsidies came from randomized trials, but these were all conducted in the US.

The meta-analysis suggested that consumers with low income increased their purchases of subsidized fruits and vegetables, although the demand response was inelastic, with the price elasticity of demand for fruit and vegetable sales estimated at −0.59. The study finding was similar to that of a previous review, which reported estimates for fruits (−0.70) and vegetables (−0.58) for the general population and included modeling studies and experimental data,^9^ and this finding was also similar to another systematic review’s estimates for fruits (−0.49) and vegetables (−0.48).^11^ One meta-analysis^7^ reported that a 10% subsidy for fruits and vegetables was associated with a 14% increase in their consumption, suggesting a much higher price elasticity of demand of −1.40, potentially an upper bound estimate. Moreover, a US Department of Agriculture study^78^ that targeted US households with a low income estimated more modest results, in line with the findings from the present study, suggested that a 10% fruit and vegetable subsidy could increase fruit consumption by 2.1% to 5.2% and vegetable consumption by 2.1% to 4.9%.^78^ The findings of inelastic demand for fruits and vegetables indicated that subsidies for healthy foods would need to be relatively large to create substantial changes in consumer purchases and ultimately improve the diet and health of a population.

Currently, data on consumption outcomes of subsidies for fruits and vegetables were inconclusive, with the pooled estimates indicating no significant changes. Yet, unlike data on sales outcomes, these consumption results were based on fewer studies that were mostly low quality. Future evaluations of the outcomes of subsidies should focus specifically on measuring consumption changes in both children and adults using statistically powered analytic samples and more complete dietary assessments, such as 24-hour dietary recalls. In addition, we found that the outcomes of fruit and vegetable subsidies had high heterogeneity, especially for sales (eTable 3 in the Supplement). This finding could be owing to large variation in study designs and quality and the differences in subsidy designs across regions (eg, type and rate). As more research becomes available, future systematic reviews and meta-analyses should assess the effectiveness of specific food taxes and subsidies across designs, food products covered, and subsidy jurisdictions.

We found convincing evidence that food taxes were associated with higher prices and reduced sales of taxed products, but a small number of available studies precluded the conduct of a meta-analysis. The existing food tax studies did not assess consumption, diet, and health. The limited evidence to date does not show any significant changes in BMI or NCDs after implementation of food-related fiscal policies, and no research was available for pregnancy and product change outcomes. The data were not granular enough to enable analyses of the fiscal policy outcomes for population subgroups. Granularity is particularly relevant in evaluating subsidies for populations with low income, which are diverse in aspects beyond household income. Future research should examine the heterogeneity of policy responses across subpopulations, especially groups that are most at risk for diet-related NCDs. There are limited data from the current literature to provide insights into the equity impact of fiscal policies for food products.

### Limitations

This study has some limitations. Although the study was based on a comprehensive search of the worldwide literature using established methods, we were unable to conduct a meta-analysis for multiple outcomes because of the low number of available studies, large heterogeneity across measures, or lack of statistical testing or missing data in some studies. Given that almost all subsidies were intended for populations with low income, the generalizability of the subsidy results to the general population is unknown. Future systematic reviews of more studies of implemented taxes and subsidies are needed to strengthen the conclusions.

## Conclusions

The systematic review and meta-analysis of implemented food taxes and subsidies worldwide found conclusive evidence that fruit and vegetable subsidies to populations with low income were associated with increased sales, whereas food taxes were associated with higher prices and reduced sales. Further research on food taxes and subsidies is needed to understand their implications for consumption, diet, and health outcomes.

## Notes

Supplement. **eAppendix 1.** Search Terms and Search Results **eAppendix 2.** Computation of Price Elasticity of Demand **eTable 1.** Quality Assessment of Fiscal Policy Studies **eTable 2.** Characteristics of Studies **eTable 3.** Sources of Heterogeneity (Tau^2^) for 3-Level Random Effects Models **eFigure.** Publication Bias: Funnel Test (Left Panel) and Egger’s Regression Test Plot (Right) for Sales: Price Elasticity of Demand for Fruit and Vegetables
