# Errors in the Results Section, Table 2, and the Supplement

**DOI:** 10.1001/jamanetworkopen.2022.21711

**Published:** 2022-06-30

In the Original Investigation titled “Evaluation of Economic and Health Outcomes Associated With Food Taxes and Subsidies: A Systematic Review and Meta-analysis,”^1^ published June 1, 2022, there were errors in the Results section, Table 2, and the Supplement. In the Results section, several sentences referring to “diet-related NCDs [noncommunicable diseases]” should have referred to “undernutrition.” In Table 2 under the fruit and vegetable subsidy category, the row for diet-related NCDs was deleted, and the direction and statistical significance of estimated outcomes description for that row was added to the undernutrition row. In the Supplement, “noncommunicable disease (NCD)” in eTable 2 was changed to “undernutrition.” The article and Supplement have been corrected.^1^
