# Supplementary material for: Evaluation of Economic and Health Outcomes Associated With Food Taxes and Subsidies: A Systematic Review and Meta-analysis
Source: JAMA Netw Open. 2022 Jun 1;5(6):e2214371. doi: 10.1001/jamanetworkopen.2022.14371 (PMC9161015; doi:10.1001/jamanetworkopen.2022.14371)
Supplement: Supplement. — eAppendix 1. Search Terms and Search Results eAppendix 2. Computation of Price Elasticity of Demand eTable 1. Quality Assessment of Fiscal Policy Studies eTable 2. Characteristics of Studies eTable 3. Sources of Heterogeneity (Tau2) for 3-Level Random Effects Models eFigure. Publication Bias: Funnel Test (Left Panel) and Egger’s Regression Test Plot (Right) for Sales: Price Elasticity of Demand for Fruit and Vegetables [file jamanetwopen-e2214371-s001.pdf]

Supplemental Online Content

Andreyeva T, Marple K, Moore TE, Powell LM. Evaluation of economic and health outcomes associated with food taxes and subsidies: a systematic review and meta-analysis. *JAMA Netw Open*. 2022;5(5):e2214371. doi:10.1001/jamanetworkopen.2022.14371

eAppendix 1. Search Terms and Search Results

eAppendix 2. Computation of Price Elasticity of Demand

eTable 1. Quality Assessment of Fiscal Policy Studies

eTable 2. Characteristics of Studies

eTable 3. Sources of Heterogeneity (Tau^2) for 3-Level Random Effects Models

eFigure. Publication Bias: Funnel Test (Left Panel) and Egger's Regression Test Plot (Right) for Sales: Price Elasticity of Demand for Fruit and Vegetables

This supplemental material has been provided by the authors to give readers additional information about their work.

© 2022 Andreyeva T et al. *JAMA Network Open.*

eAppendix 1. Search Terms and Search Results

PubMed Search Terms

Concept #1: TREATMENT OR INTERVENTION

“Taxes”[MeSH] OR “Fiscal Policy”[MeSH] OR “Health Policy/economics”[MeSH] OR “Healthy Diet/economics” [MeSH] OR “Health Promotion/economics”[MeSH] OR “Consumer Behavior/economics”[MeSH] OR “Diet/economics”[MeSH] OR “Carbonated Beverages/economics”[MeSH] OR “Government Programs/economics”[MeSH] OR “Public Health/economics”[MeSH] OR tax[tiab] OR taxes[tiab] OR taxation[tiab] OR taxed[tiab] OR taxing[tiab] OR subsidy[tiab] OR subsidies[tiab] OR subsidized[tiab] OR coupon[tiab] OR coupons[tiab] OR incentive[tiab] OR incentives[tiab] OR disincentive[tiab] OR disincentives[tiab] OR discount[tiab] OR discounts[tiab] OR discounted[tiab] OR pricing[tiab] OR priced[tiab] OR “price reduction”[tiab] OR “price increase”[tiab] OR “price promotion”[tiab] OR “price promotions”[tiab] OR “price floor”[tiab] OR “price ceiling”[tiab] OR “price cap”[tiab] OR “price caps”[tiab] OR “pricing policy”[tiab] OR “pricing policies”[tiab] OR “fiscal policy”[tiab] OR “fiscal policies”[tiab] OR voucher[tiab] OR vouchers[tiab] OR rebate[tiab] OR rebates[tiab] OR levy[tiab]

AND

Concept #2: TARGETS FOR TREATMENT OR INTERVENTION

"Food"[MeSH] OR "Food and Beverages"[MeSH] OR "Beverages"[MeSH] OR “Sugars”[MeSH] OR “Fruit”[MeSH] OR “Vegetables”[MeSH] OR “Healthy Food”[MeSH] OR "Carbonated Beverages"[MeSH] OR “Dairy Products”[MeSH] OR “Meat”[MeSH] OR “Dietary Fats”[MeSH] OR “Fishes”[MeSH] OR “Seafood”[MeSH] OR "Infant formula"[MeSH] OR
“sugary drink”[tiab] OR “sugary drinks”[tiab] OR “sugary beverages”[tiab] OR “sweetened beverage”[tiab] OR “sweetened beverages”[tiab] OR “sweetened drink”[tiab] OR “sweetened drinks”[tiab] OR “sugar-sweetened beverage”[tiab] OR “sugar-sweetened beverages”[tiab] OR “sugar-sweetened drink”[tiab] OR “sugar-sweetened drinks”[tiab] OR “carbonated beverage”[tiab] OR “carbonated beverages”[tiab] OR “carbonated-beverage”[tiab] OR “carbonated-beverages”[tiab] OR “carbonated drink”[tiab] OR “carbonated drinks”[tiab] OR “carbonated-drink”[tiab] OR “carbonated-drinks”[tiab] OR “high-calorie drink”[tiab] OR “high-calorie drinks”[tiab] OR “high calorie drink”[tiab] OR “high calorie drinks”[tiab] OR “high-calorie beverage”[tiab] OR “high-calorie beverages”[tiab] OR “high calorie beverage”[tiab] OR “high calorie beverages”[tiab] OR “low-calorie drink”[tiab] OR “low-calorie drinks”[tiab] OR “low calorie drink”[tiab] OR “low calorie drinks”[tiab] OR “low-calorie beverage”[tiab] OR “low-calorie beverages”[tiab] OR “low calorie beverage”[tiab] OR “low calorie beverages”[tiab] OR “soft drink”[tiab] OR “soft drinks”[tiab] OR “soft-drink”[tiab] OR “soft-drinks”[tiab] OR “fizzy drink”[tiab] OR “fizzy drinks”[tiab] OR “fizzy-drink”[tiab] OR “fizzy-drinks”[tiab] OR “energy

drink”[tiab] OR “energy drinks”[tiab] OR “energy-drink”[tiab] OR “energy-drinks”[tiab] OR “fruit drink”[tiab] OR “fruit drinks”[tiab] OR “fruit-drink”[tiab] OR “fruit-drinks”[tiab] OR “sports drink”[tiab] OR “sports drinks”[tiab] OR “sports-drink”[tiab] OR “sports-drinks”[tiab] OR tea[tiab] OR teas[tiab] OR coffee[tiab] OR coffees[tiab] OR soda[tiab] OR sodas[tiab] OR cola[tiab] OR colas[tiab] OR “flavored water”[tiab] OR “vitamin water”[tiab] OR SSB*[tiab] OR “soda pop”[tiab] OR “chocolate milk”[tiab] OR “flavored milk”[tiab] OR juice[tiab] OR syrup*[tiab] OR

“healthy food”[tiab] OR “healthy foods”[tiab] OR “unhealthy food”[tiab] OR “unhealthy foods”[tiab] OR “nutritious foods”[tiab] OR “junk food”[tiab] OR “junk foods”[tiab] OR “energy dense food”[tiab] OR “high-calorie food”[tiab] OR “highcalorie food”[tiab] OR “high calorie food”[tiab] OR “energy dense foods”[tiab] OR “high-calorie foods”[tiab] OR “highcalorie foods”[tiab] OR “high calorie foods”[tiab] OR “low-calorie food”[tiab] OR “lowcalorie food”[tiab] OR “low calorie food”[tiab] OR “low-calorie foods”[tiab] OR “lowcalorie foods”[tiab] OR “low calorie foods”[tiab] OR "highenergy food"[tiab] OR "high energy food"[tiab] OR "high-energy food"[tiab] OR "highenergy foods"[tiab] OR "high energy foods"[tiab] OR "high-energy foods"[tiab] OR "lowenergy food"[tiab] OR "low energy food"[tiab] OR "low-energy food"[tiab] OR "lowenergy foods"[tiab] OR "low energy foods"[tiab] OR "low-energy foods"[tiab] OR “low-fat”[tiab] OR “low fat”[tiab] OR “lowfat”[tiab] OR

“fatty food”[tiab] OR “fatty foods”[tiab] OR “fattening food”[tiab] OR “fattening-food”[tiab] OR “salty food”[tiab] OR “salty-food”[tiab] OR “processed food”[tiab] OR “fattening foods”[tiab] OR “fattening-foods”[tiab] OR “salty foods”[tiab] OR “salty-foods”[tiab] OR “processed foods”[tiab] OR “takeout food”[tiab] OR “take-out food”[tiab] OR “takeaway food”[tiab] OR “take-away food”[tiab] OR “takeout foods”[tiab] OR “take-out foods”[tiab] OR “takeaway foods”[tiab] OR “take-away foods”[tiab] OR “fast food”[tiab] OR “fast-food”[tiab] OR “fast foods”[tiab] OR “fast-foods”[tiab] OR fastfood[tiab] OR “healthy diet"[tiab] OR "healthy diets”[tiab] OR "unhealthy diet"[tiab] OR "unhealthy diets"[tiab] OR

fruit[tiab] OR fruits[tiab] OR vegetable[tiab] OR vegetables[tiab] OR legumes[tiab] OR pulses[tiab] OR beans[tiab] OR lentils[tiab] OR peas[tiab] OR peanut*[tiab] OR salad[tiab] OR potato[tiab] OR potatoes[tiab] OR burger[tiab] OR candy[tiab] OR candies[tiab] OR confectionar*[tiab] OR chips[tiab] OR snack[tiab] OR snacks[tiab] OR sweet[tiab] OR sweets[tiab] OR desert[tiab] OR “ice cream”[tiab] OR biscuit*[tiab] OR cookie*[tiab] OR chocolate*[tiab] OR pizza[tiab] OR grain[tiab] OR grains[tiab] OR pasta[tiab] OR cereal[tiab] OR cereals[tiab] OR corn[tiab] OR maize[tiab] OR rice[tiab] OR wheat[tiab] OR barley[tiab] OR sorghum[tiab] OR oat[tiab] OR oats[tiab] OR oatmeal[tiab] OR rye[tiab] OR cassava[tiab] OR soybean[tiab] OR yam[tiab] OR plantain[tiab] OR millet[tiab] OR taro[tiab] OR milk[tiab] OR “low fat dairy”[tiab] OR “low-fat dairy”[tiab] OR “lowfat dairy”[tiab] OR butter[tiab] OR cheese[tiab] OR cheeses[tiab] OR margarine[tiab] OR lard[tiab] OR yogurt[tiab] OR “soybean oil”[tiab] OR “rapeseed oil”[tiab] OR “sunflower oil”[tiab] OR “olive oil”[tiab] OR “sesame oil”[tiab] OR “palm oil”[tiab] OR “coconut oil”[tiab] OR “cooking oil”[tiab] OR “vegetable oil”[tiab] OR “soya oil”[tiab] OR “peanut oil”[tiab] OR “groundnut oil”[tiab] OR fish[tiab] OR

© 2022 Andreyeva T et al. *JAMA Network Open.*

seafood[tiab] OR pollock[tiab] OR anchovy[tiab] OR tuna[tiab] OR sardine[tiab] OR mackerel[tiab] OR herring[tiab] OR cod[tiab] OR scad[tiab] OR hairtail[tiab] OR whiting[tiab] OR squid[tiab] OR threadfin[tiab] OR menhaden[tiab] OR sprat[tiab] OR crab[tiab] OR shrimp[tiab] OR pilchard[tiab] OR

meat[tiab] OR meats[tiab] OR "lean protein"[tiab] OR beef[tiab] OR poultry[tiab] OR chicken[tiab] OR pork[tiab] OR mutton[tiab] OR goat[tiab] OR egg[tiab] OR eggs[tiab] OR fats[tiab] OR "saturated fat"[tiab] OR "unsaturated fat"[tiab] OR polyunsat*[tiab] OR poly-unsat*[tiab] OR monounsat*[tiab] OR mono-unsat*[tiab] OR transfat[tiab] OR trans-fat*[tiab] OR sodium [tiab] OR salt[tiab] OR sugar[tiab] OR sugars[tiab] OR sucrose[tiab] OR isoglucose[tiab] OR fiber[tiab] OR fibre[tiab] OR "infant formula"[tiab] OR "baby food"[tiab] OR "baby foods"[tiab] OR "infant food"[tiab] OR "infant foods"[tiab] OR "toddler formula"[tiab] OR "toddler milks"[tiab]

NOT

drug[tiab] or drugs[tiab]

Search results with the terms above on 7/3/19: n=8,261
Search results with the terms above on 1/31/20: n=258
Search results with the terms above on 6/01/20: n=205

Business Source Complete / CINAHL / EconLit / PSYCInfo Search Terms

Concept #1: TREATMENT OR INTERVENTION

TI(tax OR taxes OR taxation OR taxed OR taxing OR subsidy OR subsidies OR subsidized OR coupon OR coupons OR incentive OR incentives OR disincentive OR disincentives OR discount OR discounts OR discounted OR pricing OR priced OR "fiscal policy" OR "fiscal policies" OR voucher OR vouchers OR rebate OR rebates OR levy OR "price reduction" OR "price increase" OR "price promotion" OR "price promotions" OR "price floor" OR "price ceiling" OR "price cap" OR "price caps") OR

AB(tax OR taxes OR taxation OR taxed OR taxing OR subsidy OR subsidies OR subsidized OR coupon OR coupons OR incentive OR incentives OR disincentive OR disincentives OR discount OR discounts OR discounted OR pricing OR priced OR "fiscal policy" OR "fiscal policies" OR voucher OR vouchers OR rebate OR rebates OR levy OR "price reduction" OR "price increase" OR "price promotion" OR "price promotions" OR "price floor" OR "price ceiling" OR "price cap" OR "price caps")

AND

© 2022 Andreyeva T et al. *JAMA Network Open.*

Concept #2: TARGETS FOR TREATMENT OR INTERVENTION

TI(“sugary drink” OR “sugary drinks” OR “sugary beverages” OR “sweetened beverage” OR “sweetened beverages” OR “sweetened drink” OR “sweetened drinks” OR “sugar-sweetened beverage” OR “sugar-sweetened beverages” OR “sugar-sweetened drink” OR “sugar-sweetened drinks” OR “carbonated beverage” OR “carbonated beverages” OR “carbonated-beverage” OR “carbonated-beverages” OR “carbonated drink” OR “carbonated drinks” OR “carbonated-drink” OR “carbonated-drinks” OR “high-calorie drink” OR “high-calorie drinks” OR “high calorie drink” OR “high calorie drinks” OR “high-calorie beverage” OR “high-calorie beverages” OR “high calorie beverage” OR “high calorie beverages” OR “low-calorie drink” OR “low-calorie drinks” OR “low calorie drink” OR “low calorie drinks” OR “low-calorie beverage” OR “low-calorie beverages” OR “low calorie beverage” OR “low calorie beverages” OR “soft drink” OR “soft drinks” OR “soft-drink” OR “soft-drinks” OR “fizzy drink” OR “fizzy drinks” OR “fizzy-drink” OR “fizzy-drinks” OR “energy drink” OR “energy drinks” OR “energy-drink” OR “energy-drinks” OR “fruit drink” OR “fruit drinks” OR “fruit-drink” OR “fruit-drinks” OR “sports drink” OR “sports drinks” OR “sports-drink” OR “sports-drinks” OR tea OR teas OR coffee OR coffees OR soda OR sodas OR cola OR colas OR “flavored water” OR “vitamin water” OR SSB* OR “soda pop” OR “chocolate milk” OR “flavored milk” OR juice OR syrup* OR “healthy food” OR “healthy foods” OR “unhealthy food” OR “unhealthy foods” OR “nutritious foods” OR “junk food” OR “junk foods” OR “energy dense food” OR “high-calorie food” OR “highcalorie food” OR “high calorie food” OR “energy dense foods” OR “high-calorie foods” OR “highcalorie foods” OR “high calorie foods” OR “low-calorie food” OR “lowcalorie food” OR “low calorie food” OR “low-calorie foods” OR “lowcalorie foods” OR “low calorie foods” OR "highenergy food" OR "high energy food" OR "high-energy food" OR "highenergy foods" OR "high energy foods" OR "high-energy foods" OR "lowenergy food" OR "low energy food" OR "low-energy food" OR "lowenergy foods" OR "low energy foods" OR "low-energy foods" OR “low-fat” OR “low fat” OR “lowfat” OR “fatty food” OR “fatty foods” OR “fattening food” OR “fattening-food” OR “salty food” OR “salty-food” OR “processed food” OR “fattening foods” OR “fattening-foods” OR “salty foods” OR “salty-foods” OR “processed foods” OR “takeout food” OR “take-out food” OR “takeaway food” OR “take-away food” OR “takeout foods” OR “take-out foods” OR “takeaway foods” OR “take-away foods” OR “fast food” OR “fast-food” OR “fast foods” OR “fast-foods” OR fastfood OR fruit OR fruits OR vegetable OR vegetables OR legumes OR pulses OR beans OR lentils OR peas OR peanut* OR salad OR potato OR potatoes OR burger OR candy OR candies OR confectionar* OR chips OR snack OR snacks OR sweet OR sweets OR desert OR “ice cream” OR biscuit* OR cookie* OR chocolate* OR pizza OR chips OR grain OR grains OR pasta OR cereal OR cereals OR corn OR maize OR rice OR wheat OR barley OR sorghum OR oat OR oats OR oatmeal OR rye OR cassava OR soybean OR yam OR plantain OR millet OR taro OR milk OR “low fat dairy” OR “low-fat dairy” OR “lowfat dairy” OR butter OR cheese OR cheeses OR margarine OR lard OR yogurt OR “soybean oil” OR “rapeseed oil” OR “sunflower oil” OR “olive oil” OR “sesame oil” OR “palm oil” OR “coconut oil” OR “cooking oil” OR “vegetable oil” OR “soya oil” OR “peanut oil” OR “groundnut oil” OR fish OR fishes OR seafood OR pollock OR anchovy OR tuna OR sardine OR mackerel OR herring OR cod OR scad OR hairtail OR whiting OR squid OR threadfin OR menhaden OR sprat OR crab OR shrimp OR pilchard OR meat OR meats OR “lean protein” OR

beef OR poultry OR chicken OR pork OR mutton OR goat OR egg OR eggs OR fats OR “saturated fat” OR “unsaturated fat” OR polyunsat* OR poly-unsat* OR monounsat* OR mono-unsat* OR transfat OR trans-fat* OR sodium OR salt OR sugar OR sugars OR sucrose OR isoglucose OR fiber OR fibre OR “infant formula” OR “baby food” OR “baby foods” OR "infant food" OR "infant foods" OR “toddler formula” OR “toddler milks”) OR

AB(“sugary drink” OR “sugary drinks” OR “sugary beverages” OR “sweetened beverage” OR “sweetened beverages” OR “sweetened drink” OR “sweetened drinks” OR “sugar-sweetened beverage” OR “sugar-sweetened beverages” OR “sugar-sweetened drink” OR “sugar-sweetened drinks” OR “carbonated beverage” OR “carbonated beverages” OR “carbonated-beverage” OR “carbonated-beverages” OR “carbonated drink” OR “carbonated drinks” OR “carbonated-drink” OR “carbonated-drinks” OR “high-calorie drink” OR “high-calorie drinks” OR “high calorie drink” OR “high calorie drinks” OR “high-calorie beverage” OR “high-calorie beverages” OR “high calorie beverage” OR “high calorie beverages” OR “low-calorie drink” OR “low-calorie drinks” OR “low calorie drink” OR “low calorie drinks” OR “low-calorie beverage” OR “low-calorie beverages” OR “low calorie beverage” OR “low calorie beverages” OR “soft drink” OR “soft drinks” OR “soft-drink” OR “soft-drinks” OR “fizzy drink” OR “fizzy drinks” OR “fizzy-drink” OR “fizzy-drinks” OR “energy drink” OR “energy drinks” OR “energy-drink” OR “energy-drinks” OR “fruit drink” OR “fruit drinks” OR “fruit-drink” OR “fruit-drinks” OR “sports drink” OR “sports drinks” OR “sports-drink” OR “sports-drinks” OR tea OR teas OR coffee OR coffees OR soda OR sodas OR cola OR colas OR “flavored water” OR “vitamin water” OR SSB* OR “soda pop” OR “chocolate milk” OR “flavored milk” OR juice OR syrup* OR
“healthy food” OR “healthy foods” OR “unhealthy food” OR “unhealthy foods” OR “nutritious foods” OR “junk food” OR “junk foods” OR “energy dense food” OR “high-calorie food” OR “highcalorie food” OR “high calorie food” OR “energy dense foods” OR “high-calorie foods” OR “highcalorie foods” OR “high calorie foods” OR “low-calorie food” OR “lowcalorie food” OR “low calorie food” OR “low-calorie foods” OR “lowcalorie foods” OR “low calorie foods” OR "highenergy food" OR "high energy food" OR "high-energy food" OR "highenergy foods" OR "high energy foods" OR "high-energy foods" OR "lowenergy food" OR "low energy food" OR "low-energy food" OR "lowenergy foods" OR "low energy foods" OR "low-energy foods" OR “low-fat” OR “low fat” OR “lowfat” OR “fatty food” OR “fatty foods” OR “fattening food” OR “fattening-food” OR “salty food” OR “salty-food” OR “processed food” OR “fattening foods” OR “fattening-foods” OR “salty foods” OR “salty-foods” OR “processed foods” OR “takeout food” OR “take-out food” OR “takeaway food” OR “take-away food” OR “takeout foods” OR “take-out foods” OR “takeaway foods” OR “take-away foods” OR “fast food” OR “fast-food” OR “fast foods” OR “fast-foods” OR fastfood OR fruit OR fruits OR vegetable OR vegetables OR legumes OR pulses OR beans OR lentils OR peas OR peanut* OR salad OR potato OR potatoes OR burger OR candy OR candies OR confectionar* OR chips OR snack OR snacks OR sweet OR sweets OR desert OR “ice cream” OR biscuit* OR cookie* OR chocolate* OR pizza OR chips OR grain OR grains OR pasta OR cereal OR cereals OR corn OR maize OR rice OR wheat OR barley OR sorghum OR oat OR oats OR oatmeal OR rye OR cassava OR soybean OR yam OR plantain OR millet OR taro OR milk OR “low fat dairy” OR “low-fat dairy” OR “lowfat dairy” OR butter OR cheese OR cheeses OR margarine OR lard OR yogurt OR “soybean oil” OR “rapeseed oil” OR

© 2022 Andreyeva T et al. *JAMA Network Open.*

“sunflower oil” OR “olive oil” OR “sesame oil” OR “palm oil” OR “coconut oil” OR “cooking oil” OR “vegetable oil” OR “soya oil” OR “peanut oil” OR “groundnut oil” OR fish OR fishes OR seafood OR pollock OR anchovy OR tuna OR sardine OR mackerel OR herring OR cod OR scad OR hairtail OR whiting OR squid OR threadfin OR menhaden OR sprat OR crab OR shrimp OR pilchard OR meat OR meats OR “lean protein” OR beef OR poultry OR chicken OR pork OR mutton OR goat OR egg OR eggs OR fats OR “saturated fat” OR “unsaturated fat” OR polyunsat* OR poly-unsat* OR monounsat* OR mono-unsat* OR transfat OR trans-fat* OR sodium OR salt OR sugar OR sugars OR sucrose OR isoglucose OR fiber OR fibre OR “infant formula” OR “baby food” OR “baby foods” OR "infant food" OR "infant foods" OR “toddler formula” OR “toddler milks”)

NOT

(TI(drug OR drugs) OR AB(drug OR drugs))

LIMIT TO: Academic Journals

Search results with the terms and limitations above on 7/3/19:
- Business Source Complete: n=3,816
- CINAHL: 842
- EconLit: n=2,830
- Proquest Dissertations & Theses: n=8,404
- PSYCInfo: n=1,107

Search results with the terms and limitations above on 1/31/20:
- Business Source Complete: n=133
- CINAHL: 172
- EconLit: n=153
- Proquest Dissertations & Theses: n=121
- PSYCInfo: n=60

Search results with the terms and limitations above on 6/1/20:
- Business Source Complete: n=57
- CINAHL: 49
- EconLit: n=13
- Proquest Dissertations & Theses: n=20
- PSYCInfo: n=7

Cochrane Search Terms

Concept #1: TREATMENT OR INTERVENTION

© 2022 Andreyeva T et al. *JAMA Network Open.*

((tax OR taxes OR taxation OR taxed OR taxing OR subsidy OR subsidies OR subsidized OR coupon OR coupons OR incentive OR incentives OR disincentive OR disincentives OR discount OR discounts OR discounted OR pricing OR priced OR “price promotion” OR “price promotions” OR “price floor” OR “price ceiling” OR “price cap” OR “price caps” OR “pricing policy” OR “pricing policies” OR “fiscal policy” OR “fiscal policies” OR “price reduction” OR “price increase” OR voucher OR vouchers OR rebate OR rebates OR levy OR “price response”)):ti,ab,kw

AND

Concept #2: TARGETS FOR TREATMENT OR INTERVENTION

((“sugary drink” OR “sugary drinks” OR “sugary beverages” OR “sweetened beverage” OR “sweetened beverages” OR “sweetened drink” OR “sweetened drinks” OR “sugar-sweetened beverage” OR “sugar-sweetened beverages” OR “sugar-sweetened drink” OR “sugar-sweetened drinks” OR “carbonated beverage” OR “carbonated beverages” OR “carbonated-beverage” OR “carbonated-beverages” OR “carbonated drink” OR “carbonated drinks” OR “carbonated-drink” OR “carbonated-drinks” OR “high-calorie drink” OR “high-calorie drinks” OR “high calorie drink” OR “high calorie drinks” OR “high-calorie beverage” OR “high-calorie beverages” OR “high calorie beverage” OR “high calorie beverages” OR “low-calorie drink” OR “low-calorie drinks” OR “low calorie drink” OR “low calorie drinks” OR “low-calorie beverage” OR “low-calorie beverages” OR “low calorie beverage” OR “low calorie beverages” OR “soft drink” OR “soft drinks” OR “soft-drink” OR “soft-drinks” OR “fizzy drink” OR “fizzy drinks” OR “fizzy-drink” OR “fizzy-drinks” OR “energy drink” OR “energy drinks” OR “energy-drink” OR “energy-drinks” OR “fruit drink” OR “fruit drinks” OR “fruit-drink” OR “fruit-drinks” OR “sports drink” OR “sports drinks” OR “sports-drink” OR “sports-drinks” OR tea OR teas OR coffee OR coffees OR soda OR sodas OR cola OR colas OR “flavored water” OR “vitamin water” OR SSB OR SSBs OR “soda pop” OR “chocolate milk” OR “flavored milk” OR juice OR syrup OR syrups OR “healthy food” OR “healthy foods” OR “unhealthy food” OR “unhealthy foods” OR “nutritious foods” OR “junk food” OR “junk foods” OR “energy dense food” OR “high-calorie food” OR “highcalorie food” OR “high calorie food” OR “energy dense foods” OR “high-calorie foods” OR “highcalorie foods” OR “high calorie foods” OR “low-calorie food” OR “lowcalorie food” OR “low calorie food” OR “low-calorie foods” OR “lowcalorie foods” OR “low calorie foods” OR "highenergy food" OR "high energy food" OR "high-energy food" OR "highenergy foods" OR "high energy foods" OR "high-energy foods" OR "lowenergy food" OR "low energy food" OR "low-energy food" OR "lowenergy foods" OR "low energy foods" OR "low-energy foods" OR “low-fat” OR “low fat” OR “lowfat” OR “fatty food” OR “fatty foods” OR “fattening food” OR “fattening-food” OR “salty food” OR “salty-food” OR “processed food” OR “fattening foods” OR “fattening-foods” OR “salty foods” OR “salty-foods” OR “processed foods” OR “takeout food” OR “take-out food” OR “takeaway food” OR “take-away food” OR “takeout foods” OR “take-out foods” OR “takeaway foods” OR “take-away foods” OR “fast food” OR “fast-food” OR “fast foods” OR “fast-foods” OR fastfood OR fruit OR fruits OR vegetable OR vegetables OR legumes OR pulses OR beans OR lentils OR peas OR peanut OR peanuts OR salad OR potato OR potatoes OR burger OR candy OR candies OR confectionary OR confectionaries OR chips OR snack OR snacks OR sweet OR sweets

© 2022 Andreyeva T et al. *JAMA Network Open.*

OR desert OR “ice cream” OR biscuit OR biscuits OR cookie OR cookies OR chocolate OR chocolates OR pizza OR chips OR grain OR grains OR pasta OR cereal OR cereals OR corn OR maize OR rice OR wheat OR barley OR sorghum OR oat OR oats OR oatmeal OR rye OR cassava OR soybean OR yam OR plantain OR millet OR taro OR milk OR “low fat dairy” OR “low-fat dairy” OR “lowfat dairy” OR butter OR cheese OR cheeses OR margarine OR lard OR yogurt OR “soybean oil” OR “rapeseed oil” OR “sunflower oil” OR “olive oil” OR “sesame oil” OR “palm oil” OR “coconut oil” OR “cooking oil” OR “vegetable oil” OR “soya oil” OR “peanut oil” OR “groundnut oil” OR fish OR fishes OR seafood OR pollock OR anchovy OR tuna OR sardine OR mackerel OR herring OR cod OR scad OR hairtail OR whiting OR squid OR threadfin OR menhaden OR sprat OR crab OR shrimp OR pilchard OR meat OR meats OR “lean protein” OR beef OR poultry OR chicken OR pork OR mutton OR goat OR egg OR eggs OR fats OR “saturated fat” OR “unsaturated fat” OR polyunsaturated OR poly-unsaturated OR monounsaturated OR mono-unsaturated OR transfat OR trans-fats OR sodium OR salt OR sugar OR sugars OR sucrose OR isoglucose OR fiber OR fibre OR “infant formula” OR “baby food” OR “baby foods” OR "infant food" OR "infant foods" OR “toddler formula” OR “toddler milks”)):ti,ab,kw

NOT (drug OR drugs):ti,ab,kw

Search results in Cochrane CENTRAL with the terms above on 7/3/19: n=335
Search results in Cochrane CENTRAL with the terms above on 1/31/20: n=41
Search results in Cochrane CENTRAL with the terms above on 6/1/20: n=24
Search results in Cochrane CDSR with the terms above on 7/3/19: n=6
Search results in Cochrane CDSR with the terms above on 1/31/20: n=1
Search results in Cochrane CDSR with the terms above on 6/1/20: n=1

SCOPUS Search Terms

Concept #1: TREATMENT OR INTERVENTION

TITLE-ABS (tax OR taxes OR taxation OR taxed OR taxing OR subsidy OR subsidies OR subsidized OR coupon OR coupons OR incentive OR incentives OR disincentive OR disincentives OR discount OR discounts OR discounted OR pricing OR priced OR "fiscal policy" OR "fiscal policies" OR voucher OR vouchers OR rebate OR rebates OR levy OR "price reduction" OR “price increase” OR "price promotion" OR "price promotions" OR "price floor" OR "price ceiling" OR "price cap" OR "price caps")

AND

Concept #2: TARGETS FOR TREATMENT OR INTERVENTION

TITLE-ABS ("sugary drink" OR "sugary drinks" OR “sugary beverages” OR "sweetened beverage" OR "sweetened beverages" OR "sweetened drink" OR "sweetened drinks" OR "sugar-sweetened beverage" OR "sugar-sweetened beverages" OR "sugar-sweetened drink"

© 2022 Andreyeva T et al. *JAMA Network Open.*

OR "sugar-sweetened drinks" OR "carbonated beverage" OR "carbonated beverages" OR "carbonated-beverage" OR "carbonated-beverages" OR "carbonated drink" OR "carbonated drinks" OR "carbonated-drink" OR "carbonated-drinks" OR "high-calorie drink" OR "high-calorie drinks" OR "high calorie drink" OR "high calorie drinks" OR "high-calorie beverage" OR "high-calorie beverages" OR "high calorie beverage" OR "high calorie beverages" OR "low-calorie drink" OR "low-calorie drinks" OR "low calorie drink" OR "low calorie drinks" OR "low-calorie beverage" OR "low-calorie beverages" OR "low calorie beverage" OR "low calorie beverages" OR "soft drink" OR "soft drinks" OR "soft-drink" OR "soft-drinks" OR "fizzy drink" OR "fizzy drinks" OR "fizzy-drink" OR "fizzy-drinks" OR "energy drink" OR "energy drinks" OR "energy-drink" OR "energy-drinks" OR "fruit drink" OR "fruit drinks" OR "fruit-drink" OR "fruit-drinks" OR "sports drink" OR "sports drinks" OR "sports-drink" OR "sports-drinks" OR tea OR teas OR coffee OR coffees OR soda OR sodas OR cola OR colas OR "flavored water" OR "vitamin water" OR ssb* OR "soda pop" OR "chocolate milk" OR "flavored milk" OR juice OR syrup* OR "healthy food" OR "healthy foods" OR "unhealthy food" OR "unhealthy foods" OR "nutritious foods" OR "junk food" OR "junk foods" OR "energy dense food" OR "high-calorie food" OR "highcalorie food" OR "high calorie food" OR "energy dense foods" OR "high-calorie foods" OR "highcalorie foods" OR "high calorie foods" OR "low-calorie food" OR "lowcalorie food" OR "low calorie food" OR "low-calorie foods" OR "lowcalorie foods" OR "low calorie foods" OR "highenergy food" OR "high energy food" OR "high-energy food" OR "highenergy foods" OR "high energy foods" OR "high-energy foods" OR "lowenergy food" OR "low energy food" OR "low-energy food" OR "lowenergy foods" OR "low energy foods" OR "low-energy foods" OR "low-fat" OR "low fat" OR "lowfat" OR "fatty food" OR "fatty foods" OR "fattening food" OR "fattening-food" OR "salty food" OR "salty-food" OR "processed food" OR "fattening foods" OR "fattening-foods" OR "salty foods" OR "salty-foods" OR "processed foods" OR "takeout food" OR "take-out food" OR "takeaway food" OR "take-away food" OR "takeout foods" OR "take-out foods" OR "takeaway foods" OR "take-away foods" OR "fast food" OR "fast-food" OR "fast foods" OR "fast-foods" OR fastfood OR fruit OR fruits OR vegetable OR vegetables OR legumes OR pulses OR beans OR lentils OR peas OR peanut* OR salad OR potato OR potatoes OR burger OR candy OR candies OR confectionar* OR chips OR snack OR snacks OR sweet OR sweets OR desert OR "ice cream" OR biscuit* OR cookie* OR chocolate* OR pizza OR chips OR grain OR grains OR pasta OR cereal OR cereals OR corn OR maize OR rice OR wheat OR barley OR sorghum OR oat OR oats OR oatmeal OR rye OR cassava OR soybean OR yam OR plantain OR millet OR taro OR milk OR "low fat dairy" OR "low-fat dairy" OR "lowfat dairy" OR butter OR cheese OR cheeses OR margarine OR lard OR yogurt OR "soybean oil" OR "rapeseed oil" OR "sunflower oil" OR "olive oil" OR "sesame oil" OR "palm oil" OR "coconut oil" OR "cooking oil" OR "vegetable oil" OR "soya oil" OR "peanut oil" OR "groundnut oil" OR fish OR fishes OR seafood OR pollock OR anchovy OR tuna OR sardine OR mackerel OR herring OR cod OR scad OR hairtail OR whiting OR squid OR threadfin OR menhaden OR sprat OR crab OR shrimp OR pilchard OR meat OR meats OR "lean protein" OR beef OR poultry OR chicken OR pork OR mutton OR goat OR egg OR eggs OR fats OR "saturated fat" OR "unsaturated fat" OR polyunsat* OR poly-unsat* OR monounsat* OR mono-unsat* OR transfat OR trans-

© 2022 Andreyeva T et al. *JAMA Network Open.*

fat* OR sodium OR salt OR sugar OR sugars OR sucrose OR isoglucose OR fiber OR fibre OR "infant formula" OR "baby food" OR "baby foods" OR "infant food" OR "infant foods" OR "toddler formula" OR "toddler milks")

AND NOT TITLE-ABS (drug OR drugs)

AND (LIMIT-TO (SRCTYPE , "j"))

Search results in SCOPUS with the terms above on 7/3/19: n=15,814
Search results in SCOPUS with the terms above on 1/31/20: n=1,542
Search results in SCOPUS with the terms above on 6/1/20: n=651

Standard Grey Literature Search Terms

Concept #1: TREATMENT OR INTERVENTION

(tax* OR subsid* OR coupon* OR incentive* OR discount* OR pricing OR “price promotion” OR “pricing policy” OR “fiscal policy” OR voucher* OR rebate* OR levy)

AND

Concept #2: TARGETS FOR TREATMENT OR INTERVENTION

(“sugary drinks” OR “sugar-sweetened beverages” OR “sweetened beverages” OR “soft drinks” OR soda OR “high-sugar” OR “healthy food” OR “nutritious food” OR “fatty food” OR “high-calorie” OR “energy-dense” OR “junk food” OR “unhealthy food” OR “low fat” OR sugar OR “processed food” OR “fast food” OR fruit OR vegetables OR grain OR milk OR meat OR snack OR “lean protein” OR fats OR “saturated fat” OR “infant formula” OR sodium)

Search results with the terms above on 8/17/19:
WHO’s International Clinical Trials Registry Platform: n=9
PDQ-Evidence for Informed Health Policymaking: n=56
Healthevidence.org: n=35
WHO Global Index Medicus: n=63
WorldWideScience: n=1,318

Search results with the terms above on 1/31/20:
WHO’s International Clinical Trials Registry Platform: n=1
PDQ-Evidence for Informed Health Policymaking: n=0
Healthevidence.org: n=50
WHO Global Index Medicus: n=6

© 2022 Andreyeva T et al. *JAMA Network Open.*

WorldWideScience: n=30

Search results with the terms above on 6/1/20:
WHO's International Clinical Trials Registry Platform: n=0
PDQ-Evidence for Informed Health Policymaking: n=0
Healthevidence.org: n=17
WHO Global Index Medicus: n=0
WorldWideScience: n=0

Search results with the above terms and limited to the last three years and the top 500 most relevant results:
National Bureau of Economic Research: n=500
New papers added on 1/31 with the same search terms: 548
New papers added on 6/1 with the same search terms: 33

Search results with the above terms and limited to the top 500 most relevant results:
EconPapers: n=500
New papers added on 1/31 with the same search terms: 500
New papers added on 6/1 with the same search terms: 37

Search results with the above terms and limited to the top 100 most relevant results:
Google Scholar: n=100
New papers added on 1/31 with the same search terms: 100
New papers added on 6/1 with the same search terms: 94

Search results with the second concept only on 8/17/19:
Directory of Open Access Journals: n=250
New papers added on 1/31 with the same search terms: 65
New papers added on 6/1 with the same search terms: 20

Highly Simplified Grey Literature Search Terms

"tax*" OR "subsid*" OR "coupon*" OR "incentive*" OR "discount*" OR "pricing"

Free-text Search results with the terms above on 8/17/19:
EPPI-Centre Database of Promoting Health Effectiveness Reviews (DoPHER): n=359
EPPI-Centre Trials Register of Promoting Health Interventions (TRoPHI): n=324

Free-text Search results with the terms above on 6/1/20:
EPPI-Centre Database of Promoting Health Effectiveness Reviews (DoPHER): n=25
EPPI-Centre Trials Register of Promoting Health Interventions (TRoPHI): n=2

Two Word Grey Literature AND Combo Search Terms

© 2022 Andreyeva T et al. *JAMA Network Open.*

tax and beverages
tax and drinks
tax and food
incentive and beverages
incentive and drinks
incentive and food
subsidy and beverages
subsidy and drinks
subsidy and food
pricing and beverages
pricing and drinks
pricing and food
"fiscal policy" and beverages
"fiscal policy" and drinks
"fiscal policy" and food

Search results with the terms above on 8/17/19:
SSRN eLibrary: n=1,226
New papers added on 1/31 with the same search terms: 54
New papers added on 6/1 with the same search terms: 0

Hand-Screened References

References from systematic reviews identified using the search strategy above:
n=2,109

References from papers meeting the inclusion criteria
n=90

Hand-Screened Government Websites

Reports from the World Health Organization website
n=30
Reports from the United States Department of Agriculture website
n=8
Reports from foreign government websites
n=46
Reports from other government websites
n=3

We used Covidence (https://app.covidence.org) and Mendeley (https://www.mendeley.com) as the reference management software to manage the review.

© 2022 Andreyeva T et al. *JAMA Network Open.*

eAppendix 2. Computation of Price Elasticity of Demand

In this review, in addition to examining effect size estimates of outcomes for changes in demand (e.g., sales and consumption), we also convert each demand estimate into a common metric measure known as price elasticity of demand. The price elasticity of demand measure is calculated as a percentage change in demand (sales or consumption) over a percentage change in price. To estimate the price elasticity of demand for subsidized food products, percentage change in price was calculated for each subsidy assuming a 100% pass through, so that the price change was equal to the price discount (e.g., a 50% discount or rebate would correspond to a price change of -50%).

© 2022 Andreyeva T et al. *JAMA Network Open.*

eTable 1. Quality Assessment of Fiscal Policy Studies

	Question	Scoring System (Yes = 1.0, Partial = 0.5, No = 0)
Control group	1. Does the study compare outcomes in an appropriate control group?	Yes = Control group (e.g., not impacted by policy) or counterfactual used.
		No = Uncontrolled study (no control group or counterfactual).
Measures	2. Does the study measure outcomes objectively?	Yes = Objective measures: sales (scanner data), purchases (receipts of purchases), prices (scanner data), measured body weight/height, health measurements (e.g., blood pressure), 24-hour dietary intake recall, government/ administrative records.
		Partial = Data recorded by raters or households, e.g., households recording purchases without receipts.
		No = Participant self-reported data: self-reported BMI, food frequency surveys, other surveys with self-reported data.
Follow Up	3. Does the study assess outcomes in the same people/ stores before and after the intervention?	Yes = Longitudinal design: same people/households/stores at baseline and follow-up.
		No = Not the same people/households/stores, e.g., repeated cross-sections or longitudinal data with high attrition rates (>25%).
Duration	4. How many data points does the study examine before and after the intervention?	Yes = The study has ≥3 data points before and after the intervention.
		Partial = The study meets the above criteria for either a pre- or post-intervention, but not both.
		No = The study has 1-2 observations per pre- and post-assessment.
Sample	5. Does the study use a representative sample of the population affected by the policy?	Yes = Representative data of the affected population, e.g., random population sampling.
		Partial = Somewhat representative of the affected population, includes scanner household consumer panels.
		No = Not fully representative or convenience sample, e.g., limited to only one store type or one chain, select list of products or brands or group of customers.
Sample	6. Does the study have a sufficient sample size?	Yes = Sample size or power analyses provided and shows sufficient sample size.
		No = No information provided or individual sample sizes appear insufficient; e.g., n<500 individuals per site/time period or store audits n<50 per site/time period.

© 2022 Andreyeva T et al. *JAMA Network Open.*

Confounding	7. Does the study adjust adequately for likely confounders?	Yes = If an ITS then it adjusted/removed the effect of existing time trends, seasonality, and economic fluctuations. If different people are in comparison groups, then also adjusts for demographic differences, such as age and socioeconomic status.
		Partial = Some of these factors were adjusted for.
		No = None of these factors were adjusted for.
**Total Score:** Sum scores on all measures (range 0 to 7)<br>Low quality score ≤3.0<br>Medium quality score 3.5-4.5<br>High quality score ≥5.0		

© 2022 Andreyeva T et al. *JAMA Network Open.*

eTable 2. Characteristics of Studies

Source	Location	Tax type (taxed products)	Subsidy type, (products)	Outcome(s)	Study design	Source/Type of data	Population [a]	Study quality	Peer-reviewed[b]
Aguilar et al,[24] 2019	Mexico	8% ad valorem excise tax (nonessential energy-dense foods)		Price changes, sales/direct, sales/ substitution	ITS with counterfactual	Scanner: Kantar Worldpanel	All	High	No[b]
Aguilera Aburto et al,[25] 2017	Mexico	8% ad valorem excise tax (nonessential energy-dense foods)		Price changes	Uncontrolled ITS	Survey: INEGI	All	High	Yes
An and Sturm,[26] 2017	South Africa		10% and 25% cash-back rebate (healthy foods)	Consumption/direct, consumption/ substitution	Cross-sectional with instrumental variable	Primary: survey	Members of private health insurance plan	Low	Yes
An et al,[27] 2013	South Africa		10% and 25% cash-back rebate	Consumption/direct, consumption/ substitution, BMI	Cross-sectional with random	Primary: survey	Members of private health insurance plan	Low	Yes

© 2022 Andreyeva T et al. *JAMA Network Open.*

			(healthy foods)		and fixed effects				
Andreyeva and Luedicke,[28] 2015	US		$6-10/month vouchers (fruits and vegetables)	Sales/direct	Uncontrolled ITS	Scanner: one grocery chain	FA recipients (WIC)	High	Yes
Anliker et al,[29] 1992	US		Cash-value voucher (fruits and vegetables)	Consumption/direct	Uncontrolled before and after	Primary: survey	FA recipients (WIC)	Low	Yes
Atoloye,[30] 2019	US		50% discount (fruits and vegetables)	Consumption/direct	Uncontrolled before and after	Primary: survey	FA recipients (SNAP)	Low	No
Bartlett et al,[31] 2014	US		30% discount (fruits and vegetables)	Consumption/direct, consumption/ substitution, dietary intake, sales/direct	Randomized trial	Primary	FA recipients (SNAP)	High	No
Batis et al,[32] 2016	Mexico	8% ad valorem excise tax (nonessential energy-dense foods)		Sales/direct, sales/substitution	ITS with counterfactual	Scanner: Nielsen Consumer Panel	All	High	Yes

© 2022 Andreyeva T et al. *JAMA Network Open.*

Bíró,[33] 2015	Hungary	Volume-based excise tax (unhealthy and nonessential foods)		Sales/direct, sales/substitution	Controlled before and after	Scanner: Hungarian consumer panel	All	High	Yes
Black et al,[34] 2013	Australia		Discounted allotment/weekly box (fruits and vegetables)	Consumption/direct, dietary intake, NCDs	Uncontrolled before and after	Primary: survey	Disadvantaged Aboriginal children	Low	Yes
Black et al,[35] 2013	Australia		Discounted allotment/weekly box (fruits and vegetables)	BMI, undernutrition	Uncontrolled before and after	Primary: survey	Disadvantaged Aboriginal children	Low	Yes
Black et al,[36] 2014	Australia		Discounted allotment/weekly box (fruits and vegetables)	NCDs	Uncontrolled before and after	Primary: survey	Disadvantaged Aboriginal children	Low	Yes

© 2022 Andreyeva T et al. *JAMA Network Open.*

Bødker et al,[37] 2015	Denmark	DKK 16/kg of saturated fat excise tax (saturated fat)		Sales/direct	Uncontrolled before and after	Scanner: Nielsen retail	All	Medium	Yes
Bonilla-Chacin et al,[38] 2016	Mexico	8% ad valorem excise tax (nonessential energy-dense foods)		Price changes	Uncontrolled before and after	Survey: INEGI	All	Medium	No
				Sales/direct, sales/ substitution		Survey: ENIGH		Low	
Bowling et al,[39] 2016	US		Discount and cash-value voucher (fruits and vegetables)	Consumption/direct, consumption/ substitution	Uncontrolled before and after	Primary: survey	FA recipients (SNAP, WIC)	Low	Yes
Chakrabarti et al,[40] 2018	India		Discount (staple foods/pulses)	Consumption/direct, sales/direct, dietary intake	Controlled before and after	Household surveys: Consumer Expenditure Survey, Village Dynamics in South Asia panel	Low-income populations	Medium	Yes

© 2022 Andreyeva T et al. *JAMA Network Open.*

Chakrabarti et al,[41] 2019	India		Discount (staple foods/ fortified wheat flour)	Undernutrition	Controlled before and after	Household survey: Indian District Level Health survey	Pregnant women, low income	Medium	Yes
Colchero et al,[42] 2017	Mexico	8% ad valorem excise tax (nonessential energy-dense foods)		Price changes	Uncontrolled before and after	Primary: store audits	All	Low	Yes
Durward et al,[43] 2019	US		50% discount (fruits and vegetables)	Consumption/direct	Uncontrolled before and after	Primary: survey	FA recipients (SNAP)	Low	Yes
ECSIPC,[44] 2014	Denmark	DKK 16/kg of saturated fat excise tax (saturated fat)		Price changes, sales/direct	Uncontrolled before and after	Database: Euromonitor Passport	All	Low	No
	Denmark	Weight-based excise tax (ice cream,							

© 2022 Andreyeva T et al. *JAMA Network Open.*

		chocolate, sweets)							
	Finland	Weight-based excise tax (confectionary, ice cream)							
	Hungary	Weight-based excise tax (unhealthy and nonessential food)							
French et al,[45] 2017	US		30% discount (fruit and vegetables)	Sales/direct, sales/substitution	Randomized trial	Primary	Low-income adults	High	Yes
Gordes,[46] 2016	US	State sales tax (candy, snacks)		BMI	Cross-sectional with fixed effects	Survey: BRFSS	Adults	Low	Yes
Griffith et al,[47] 2018	UK		Cash-value voucher (fresh fruit and vegetables, milk)	Sales/direct	Controlled before and after	Scanner: Kantar Worldpanel	Low-income families with young children	High	Yes

© 2022 Andreyeva T et al. *JAMA Network Open.*

Grindal et al,[48] 2016	US		30% discount (fruits and vegetables)	Sales/direct	Randomized trial	Primary	FA recipients (SNAP)	High	Yes
Guerrero-Lopez et al,[49] 2017	Mexico	8% ad valorem excise tax (nonessential energy-dense foods)		UC (unemployment)	Uncontrolled ITS	Survey: industry EMIM	All	High	Yes
Harnack et al,[50] 2016	US		30% discount (fruits and vegetables)	Consumption/direct, consumption/substitution, BMI, dietary intake	Randomized trial	Primary	Low-income adults	High	Yes
Henderson,[51] 2020	US		50% discount (fruits and vegetables)	Price changes, sales/direct	Controlled before and after	Primary: survey	FA recipients (SNAP)	Medium	No
Herman et al,[52] 2008	US		Cash-value voucher (fruits and vegetables)	Consumption/direct	Controlled before and after	Primary: survey	FA recipients (WIC)	Low	Yes
Hoy,[53] 2017	Colorado, US	State sales tax (candy)		Sales/direct, sales/substitution	Controlled before and after	Scanner: Nielsen	All	High	No

© 2022 Andreyeva T et al. *JAMA Network Open.*

						Consumer Panel			
Jensen and Smed,[54] 2013	Denmark	DKK 16/kg of saturated fat excise tax (saturated fat)		Price changes, sales/direct	Uncontrolled ITS	Scanner: GfK Panel Services Denmark	All	High	Yes
Jensen et al,[55] 2016	Denmark	DKK 16/kg of saturated fat excise tax (saturated fat)		Price changes, sales/direct	Uncontrolled before and after	Scanner: Coop Danmark (5 grocery chains)	All	High	Yes
Klerman et al,[56] 2014	US		30% discount (fruits and vegetables)	Consumption/direct	Randomized trial	Primary	FA recipients (SNAP)	High	Yes
Lindsay et al,[57] 2013	US		50% discount (fruits and vegetables)	Consumption/direct	Uncontrolled before and after	Primary: survey	FA recipients (SNAP, WIC)	Low	Yes
Moran et al,[58] 2019	US		50% discount (fruits and vegetables)	Consumption/ direct	Randomized trial	Primary	Low-income families with children	Low	Yes
				Sales/direct, sales/substitution		Scanner: one		High	

© 2022 Andreyeva T et al. *JAMA Network Open.*

						grocery chain			
Moreno Neri et al,[59] 2016	Mexico	8% ad valorem excise tax (nonessential energy-dense foods)		Sales/direct, UC (employment)	Uncontrolled before and after	Primary: survey	Bakeries	Low	No
Nipers et al,[60] 2019	Latvia		Reduced value added tax (fruits and vegetables)	Price changes	Controlled before and after	Primary: survey	All	Medium	Yes
Oaks,[61] 2005	Maine, US	State sales tax (snacks)		BMI	Controlled ITS	Survey: BRFSS	Adults	Medium	No
Olsho et al,[62] 2015	US		40% discount (fruits and vegetables)	Consumption/direct	Controlled before and after	Survey: community health survey	New York City residents	Low	Yes
Olsho et al,[63] 2016	US		30% discount (fruits and vegetables)	Consumption/direct, consumption/substitution, dietary intake	Randomized trial	Primary	FA recipients (SNAP)	High	Yes
Pedraza et al,[64] 2018	Mexico	8% ad valorem excise tax (nonessenti		Sales/direct, sales/substitution	Uncontrolled ITS	Scanner: Nielsen Consumer Panel	All	Medium	Yes

© 2022 Andreyeva T et al. *JAMA Network Open.*

		al energy-dense foods)							
Phipps et al,[65] 2015	US		50% discount (fruits and vegetables)	Sales/direct	Randomized trial	Primary	Low-income families with children	High	Yes
Polacsek et al,[66] 2018	US		50% discount (fruits and vegetables)	Sales/direct	Randomized trial	Primary	Low-income families with children	High	Yes
Rummo et al,[67] 2019	US		50% discount (fruits and vegetables)	Sales/direct, sales/substitution	Controlled before and after	Scanner: one grocery chain	FA recipients (SNAP)	High	Yes
Salgado and Ng,[68] 2019	Mexico	8% ad valorem excise tax (nonessential energy-dense foods)		Price changes	Uncontrolled before and after	Scanner: Nielsen Consumer Panel	All	High	Yes
Savoie-Roskos et al,[69] 2016	US		50% discount (fruits and vegetables)	Consumption/direct	Uncontrolled before and after	Primary: survey	Adults	Low	Yes

© 2022 Andreyeva T et al. *JAMA Network Open.*

Smed et al,[70] 2016	Denmark	DKK 16/kg of saturated fat excise tax (saturated fat)		Sales/direct, sales/substitution	Uncontrolled ITS	Scanner: GfK Panel Services Scandinavia	All	High	Yes
Smith,[71] 2017	US		\$6-10/month cash-value voucher (fruits and vegetables)	Consumption/direct	Controlled before and after	Primary: survey	FA recipients (WIC)	Low	No
Steele-Adjognon and Weatherspoon,[72] 2017	US		50% discount (fruits and vegetables)	Sales/direct	Controlled before and after	Scanner: one grocery chain	FA recipients (SNAP)	High	Yes
Sturm et al,[73] 2013	South Africa		10% and 25% cash-back rebate (healthy foods)	Sales/direct, sales/substitution	Controlled before and after	Scanner: credit card purchases Pick N Pay	Members of private health plan	High	Yes
Taillie et al,[74] 2017	Mexico	8% ad valorem excise tax (nonessential energy-		Sales/direct, sales/substitution	ITS with counterfactual	Scanner: Nielsen Consumer Panel	All	High	Yes

© 2022 Andreyeva T et al. *JAMA Network Open.*

		dense foods)							
Vericker et al,[75] 2019	US		50% discount (fruits and vegetables)	Sales/direct, consumption/direct	Controlled before and after	Primary: survey	FA recipients (SNAP)	Medium	No
Wilde et al,[76] 2016	US		30% discount (fruits and vegetables)	Sales/direct	Randomized trial	Primary	FA recipients (SNAP)	Medium	Yes
Zenk et al,[77] 2014	US		$6-10/month vouchers (fruits and vegetables)	Price changes	Uncontrolled before and after	Primary: store audits	All	Medium	Yes

Abbreviations: INEGI, Mexico's Statistics Institute (*Instituto Nacional de Estadística y Geografía*, Spanish); BMI, body mass index; FA, food assistance; WIC, Special Supplemental Nutrition Assistance Program for Women, Infants, and Children; SNAP, Supplemental Nutrition Assistance Program; CBA, controlled before and after; ITS, interrupted time series; NCD, non-communicable disease; ENIGH, National Income and Expenditure Household Survey (in Spanish); BRFSS, Behavioral Risk Factor Surveillance System; UC, unintended consequences; EMIM, Monthly Survey of Manufacturing Industry.

Notes: An ITS design is defined as a study design that uses longitudinal data and has three or more post-treatment periods. An ITS was further classified as a controlled ITS, ITS with a counterfactual, and uncontrolled ITS. A controlled ITS used non-treated sites as a comparison; an ITS with a counterfactual used a counterfactual that was not untreated sites, for example, the pre-treatment trend or untaxed foods; an uncontrolled ITS did not have any type of comparison group. A before and after study used longitudinal data and made comparisons in two time periods: before and after tax implementation. Before and after studies were further classified as

© 2022 Andreyeva T et al. *JAMA Network Open.*

controlled before and after, and uncontrolled before and after. The criteria for these classifications are the same as for the ITS study designs.

[a] Not reporting #s of participants due to differences in the type of participants across studies (e.g., products, households, stores).
[b] Published in peer-reviewed literature since June 1, 2020.

© 2022 Andreyeva T et al. *JAMA Network Open.*

eTable 3. Sources of Heterogeneity (Tau^2) for 3-Level Random Effects Models

	Source of τ2 (95% CI)	
Outcome	Between study	Within study
Fruit and vegetable consumption	0.112 (0.000, 0.580)	0.001 (0.000, 0.209)
Fruit and vegetable sales	0.334 (0.000, 0.762)	0.034 (0.000, 0.457)

© 2022 Andreyeva T et al. *JAMA Network Open.*

eFigure. Publication Bias: Funnel Test (Left Panel) and Egger's Regression Test Plot (Right) for Sales: Price Elasticity of Demand for Fruit and Vegetables

Egger's test is significant (*P*<.05).

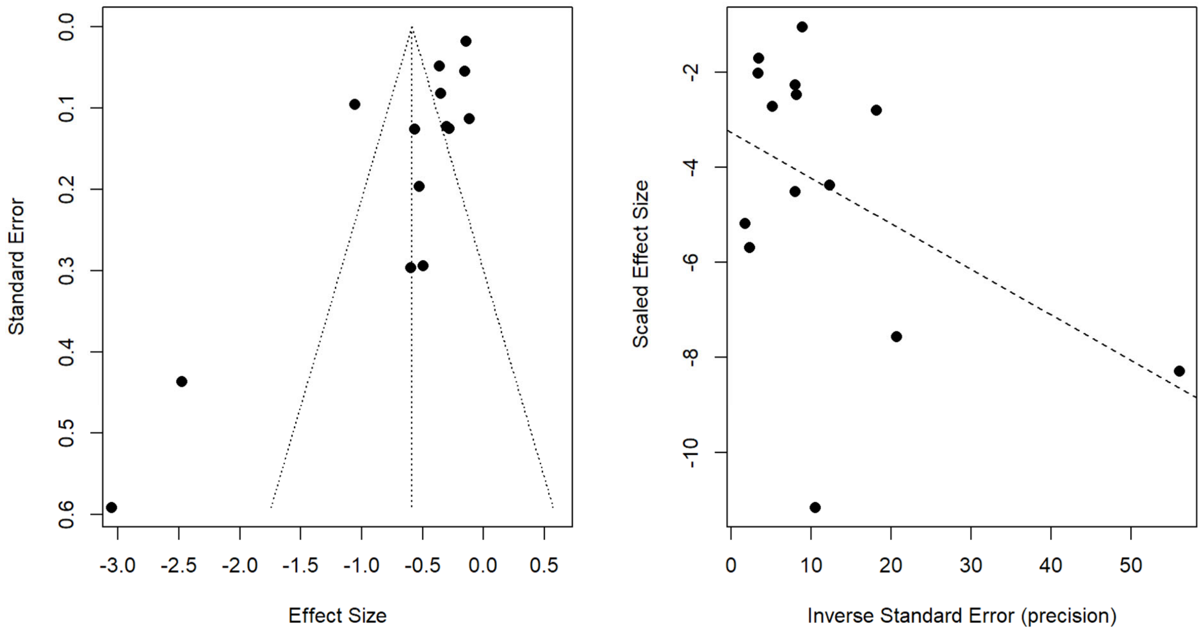

© 2022 Andreyeva T et al. *JAMA Network Open.*
